# Supplementary material for: A hybrid stochastic-deterministic approach to explore multiple infection and evolution in HIV
Source: PLoS Comput Biol. 2021 Dec 22;17(12):e1009713. doi: 10.1371/journal.pcbi.1009713 (PMC8730440; doi:10.1371/journal.pcbi.1009713)
Supplement: S1 Text — (PDF) [file pcbi.1009713.s001.pdf]

**A hybrid stochastic-deterministic approach to explore multiple infection and evolution in HIV**

# S1 Text

**Jesse Kreger[1,2,*], Natalia L. Komarova[2] and Dominik Wodarz[3,2]**

1) Department of Quantitative and Computational Biology, University of Southern California, Los Angeles, California, USA

2) Department of Mathematics, University of California Irvine, Irvine, California, USA

3) Department of Population Health and Disease Prevention Program in Public Health Susan and Henry Samueli College of Health Sciences, University of California, Irvine, California, USA

*jessekre@usc.edu

---

## Contents

**1 Details of mathematical modeling** **1**
- 1.1 Determination of model parameters . . . . . 1
- 1.2 The effect of free virus . . . . . 2
- 1.3 The effect of $N$, the maximum multiplicity of infection . . . . . 3
- 1.4 Derivation of $R_0$ . . . . . 4

**2 Details of the hybrid method** **5**
- 2.1 Description of algorithm . . . . . 5
- 2.2 Classification of reactions . . . . . 6
- 2.3 Choosing a size threshold $\mathcal{M}$ . . . . . 7
- 2.4 Comparison with tau-leaping method . . . . . 7
- 2.5 Comparison with piecewise deterministic Markov processes . . . . . 9

**3 Comparison of the ODE and stochastic/hybrid simulations in the context of infection dynamics** **9**

**4 Impact of multiple infection and synaptic transmission on mutant evolution: additional results** **13**

# 1 Details of mathematical modeling

## 1.1 Determination of model parameters

Based on experimental evidence, we estimate the kinetic parameters in the model. Assume for simplicity that all virus strains are neutral with $F = 1$. It follows that the basic reproductive number is $R_0 = \frac{\lambda(\beta+\gamma)}{ad}$ (see Section 1.4 for a general derivation). The infection steady state for infected cells is $\frac{\lambda}{a} - \frac{d}{(\beta+\gamma)}$ and the virus free steady state for uninfected cells is $\frac{\lambda}{d}$.

We have an infection steady state value of $3.1 \times 10^7$ infected cells [1]. Assuming that the death rate of infected cells is approximately 2.2 days [2], we assume that $a = 0.45$ days$^{-1}$. Further, we

set $R_0 = 8$ [3, 4]. Finally, we introduce parameter $\eta$ such that we have a total body population of $10^\eta$ lymphocytes at virus free steady state. Then we have the system of equations

$$
\begin{aligned}
3.1 \times 10^7 &= \frac{\lambda}{a} - \frac{d}{(\beta+\gamma)}, && (1)\\
8 &= \frac{\lambda(\beta+\gamma)}{ad}, && (2)\\
10^\eta &= \frac{\lambda}{d}. && (3)
\end{aligned}
$$

These equations uniquely imply that $\lambda \approx 1.59 \times 10^7$ days$^{-1}$, $d = \lambda \times 10^{-\eta}$ days$^{-1}$, and $\beta + \gamma = 3.6 \times 10^{-\eta}$ days$^{-1}$. We assume $S = 3$ [5]. Further, we choose $\eta = 9$ [1], and therefore have $d \approx 0.0159$ days$^{-1}$. For initial conditions, we assume uninfected steady state and a single infected cell (infected with the wild-type) [6], that is $x_{0,0,\ldots,0} = \frac{\lambda}{d}$ and $x_{1,0,\ldots,0} = 1$.

At these parameters, triple and further mutant strains are negligible at peak infection, that is on average less than half a cell is infected with these strains when the number of infected cells is maximized (around seven days into infection). After peak infection, the system will settle to an equilibrium. These patterns can be seen by running the model with triple mutation first to peak infection, and then many days past peak infection.

## 1.2 The effect of free virus

In the model described in the main text, we do not explicitly include free virus equations, as we assume that the free virus population is at quasi-steady state and therefore that the rate of free virus transmission is proportional to the interactions between infected and target cells. This is a reasonable assumption as the time-scale of virus spread is long compared to the very quick time-scale of the free virus particles (denoted by $v$). We have verified this for our model by comparing simulations with and without the explicit free virus equations, which is described below.

A simple example of our model with explicit equations for the free virus population is given below, and includes only free virus transmission, one virus strain, and single infection:

$$
\begin{aligned}
\dot{x} &= \lambda - \bar{\beta} x v - dx, && (4)\\
\dot{y} &= \bar{\beta} x v - ay, && (5)\\
\dot{v} &= ky - uv, && (6)
\end{aligned}
$$

where the population of free virus particles is given by $v$, $\bar{\beta}xv$ is the rate at which uninfected cells become infected, and new virus particles are produced from infected cells at rate $ky$ and die at rate $uv$. To parameterize this model we used estimates for $k$ and $u$ given in [2, 7], values are given in the caption of Fig S1. In particular, we assume $5 \times 10^4$ virions are produced by an infected cell during its lifespan, which implies $\frac{k}{a} = 5 \times 10^4$, and thus that $k = 2.25 \times 10^4$ virions per cell per day (as we assume that the death rate of infected cells is approximately 2.2 days [2]). While estimates for viral clearance rates vary (and have been shown to be different in different tissues in the body), since most virus production takes place in the lymphoid tissue we use $u = 500$ per day (see Fig 1A and the discussion in [7]).

Fig S1 compares the ODE prediction in Fig 1A in the main text to the corresponding system including explicit free virus equations; numerical values are also included in Table A. The blue dots represent the deterministic prediction for the number of cells infected with the mutant strain when the total number of infected cells has reached $10^4$ (and are the same as the blue dots in Fig 1A in the main text), in the absence of the explicit free virus equations. The red stars represent the same quantity in the presence of the free virus equations. Specifically, as in Fig 1A in the main text,

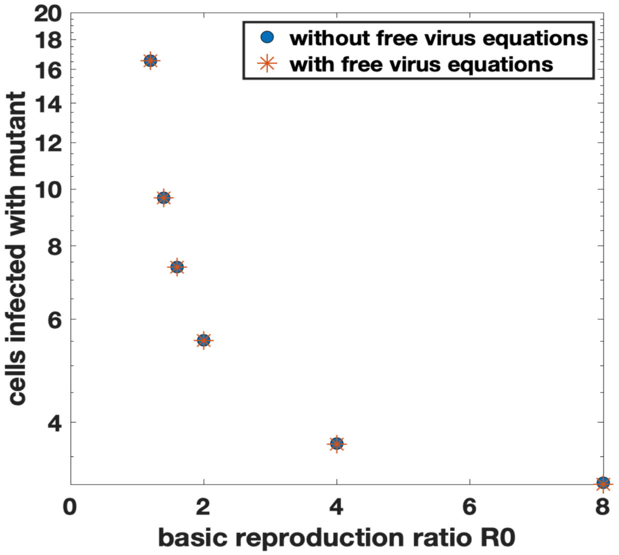


Fig S1: A comparison of the ODE prediction in Fig 1A in the main text to the corresponding system including explicit free virus equations; numerical values are also included in Table A. The blue dots represent the deterministic prediction for the number of cells infected with the mutant strain when the total number of infected cells has reached $10^4$ (and are the same as the blue dots in Fig 1A in the main text), in the absence of the explicit free virus equations. The red stars represent the same quantity in the presence of the free virus equations. The parameters are $F_{\text{wild-type}} = 0.9,\ N = 3,\ \mu = 3 \times 10^{-5},\ \lambda = 1.59 \times 10^{7},\ \beta = 4 \times 10^{-9},\ \gamma = 0,\ k = 2.25 \times 10^{4},\ u = 500$, and $d = 0.016$. The infected cell death rate $a$ is adjusted to achieve the required $R_0$.

we implement the single mutation model (only two virus strains) and consider a neutral mutant, $F_{\text{mutant}} = 0.9$. We have $R_0 = \frac{\lambda\left(\beta F + \gamma(1-(1-F)^S)\right)}{ad}$ for the model without free virus equations, and use $\bar{\beta} = \frac{\beta u}{k}$ for the model with explicit free virus equations.

| $R_0$ | 1.2 | 1.4 | 1.6 | 2 | 4 | 8 |
|---|---|---|---|---|---|---|
| % difference | 0.008 | 0.026 | 0.043 | 0.077 | 0.238 | 0.513 |

Table A : Numerical values for Fig S1, comparing deterministic predictions for the number of cells infected with the mutant with/without free virus equations.

We find that if $R_0$ is closer to 1, then the ODE predictions with/without explicit free virus equations are almost exactly the same (because the time-scale of virus spread is so long compared with the time-scale of the free virus, $v$). For HIV we have $R_0 = 8$, and the ODE predictions with explicit free virus equations result in a slightly different mean (about a 0.5% difference), because the virus does not quite catch up with $y$ (the infected cells) on the time-scale of the spread. This demonstrates that the assumption that the free virus population is at quasi-steady state is reasonable and therefore we assume that the rate of free virus transmission is proportional to the interactions between infected and target cells.

## 1.3 The effect of $N$, the maximum multiplicity of infection

The parameter $N$ (see main text, Section 2.1) represents the maximum multiplicity of infection, which is the maximum number of viral copies a cell can be infected with. It turns out (see e.g. Fig S2) that for parameters relevant for this study, most cells do not become infected with more than 11 copies of virus by the time of peak infection (in the case of only free virus transmission). Similarly, for simulations with only synaptic transmission, most cells do not become infected with more than 25 copies of virus by the time of peak infection. Therefore, for simulations with only free

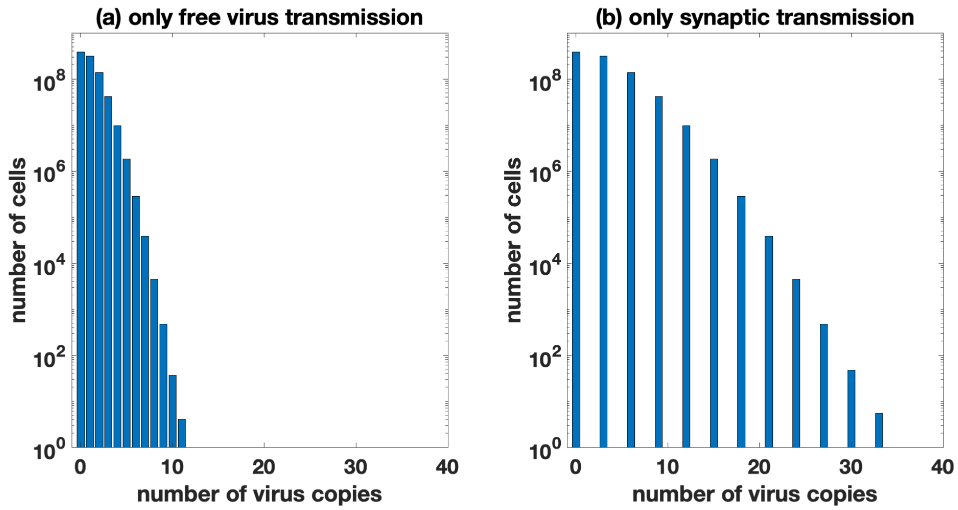


Fig S2: Histograms of the multiplicity of infection near peak infection. the horizontal axis represents the average number of cells infected with the given number of viral copies, A for only free virus transmission, B for only synaptic transmission. Fig S8 also shows histograms for the average number of cells infected with the given number of viral copies for half free virus transmission and half synaptic transmission. The vertical axis is the average number of cells that are infected with different numbers of viral copies near peak infection. Infected with zero copies corresponds to the uninfected cells. Histograms were averaged over $10^2$ hybrid simulations with size threshold $\mathcal{M} = 50$. Simulations are stopped when the infected cell population is near peak infection ($5 \times 10^8$ cells). Parameters are $\beta + \gamma = c = 3.6 \times 10^{-9}$, $\mu = 3 \times 10^{-5}$, $\lambda = 1.59 \times 10^7$, $a = 0.45$, and $d = 0.016$, and maximum multiplicity of infection $N$ is set to be large enough such that no cells reach this threshold.

virus transmission, where only one virus copy is transmitted per infection event, we use maximum multiplicity of infection $N = 11$ and for simulations with only synaptic transmission, where $S$ virus copies are transmitted per infection event, we use maximum multiplicity of infection $N = 25$. Increasing $N$ in either case (free virus only versus synaptic only) will have no effect, as almost all cells do not reach this threshold. We use a smaller $N$ for simulations with only free virus transmission for faster computational speed.

### 1.4 Derivation of $R_0$

Let $x_i$ denote the population of cells infected with $i$ copies of virus. Let $Z = \sum_{i=1}^{N} x_i$ be the total number of infected cells. Assume we have free virus transmission with rate $\beta$ and synaptic transmission with rate $\gamma$ and that $S \in \mathbb{Z}^+$ is the attempted number of viral copies transferred per synaptic transmission event. Assume the strain we initialize infection with (only one cell initially infected) has fitness $F \leq 1$ (this is the probability that an individual virus copy goes through during an infection event). For simplicity of writing down the equations, assume there is no mutation $\mu = 0$ and only one strain. Then the ODEs can be written as follows:

$$\dot{x}_0 = \lambda - \beta Z F x_0 - \gamma Z[1 - (1-F)^S]x_0 - dx_0, \tag{7}$$

$$\dot{x}_i = \beta Z F (x_{i-1} - x_i) + \gamma Z \sum_{j=1}^{S} \binom{S}{j} F^j (1-F)^{S-j}(x_{i-j} - x_i) - a x_i \tag{8}$$

$$\dot{x}_N = \beta Z F (x_{N-1}) + \gamma Z \sum_{j=1}^{S} \binom{S}{j} F^j (1-F)^{S-j} x_{N-j} - a x_N \tag{9}$$

where terms with a negative index are defined to be 0 and infection does not go through if it results in a cell being infected with more than $N$ copies of virus. Also note here that for the uninfected

cell population we make the simplification $\sum_{j=1}^{S} \binom{S}{j} F^j (1-F)^{S-j} x_0 = [1-(1-F)^S] x_0$.

To obtain the steady states we set $\dot{x}_i = 0$ for $i = 0, 1, \ldots, N$. The infection free steady state occurs when $x_i = 0$ for $i = 1, 2, \ldots, N$. In this case the differential equations for $i = 0, 1, \ldots, N$ give us no information and by solving for $x_0$ using the uninfected cell population ODE we get

$$x_0 = \frac{\lambda}{d}, \; Z = 0.$$

To get the infection steady state we sum all ODEs for the infected cell subpopulations ($i > 0$). Here, note that most of the infection terms cancel out. Using the uninfected cell population ODE as well, we have two equations with two variables ($x_0$ and $Z$). By solving these we get

$$x_0 = \frac{a}{\beta F + \gamma(1-(1-F)^S)}, \; Z = \frac{\lambda}{a} - \frac{d}{\beta F + \gamma(1-(1-F)^S)}.$$

Through standard linear stability analysis, we determine that the infection steady state is stable (real part of all eigenvalues negative), as long as $R_0 > 1$, where the basic reproductive number $R_0$ is

$$R_0 = \frac{\lambda\big(\beta F + \gamma(1-(1-F)^S)\big)}{ad}.$$

The infection-free steady state is stable if $R_0 < 1$ and that the infection steady state is stable if $R_0 > 1$.

If different strains are present and have different fitness, then different reproductive numbers $R_0$ can be found, each associated with a different strain. In the present context, as we initiate infection with a single cell infected with only the wild-type strain, we set the basic reproductive number $R_0$ to be that of the wild type virus, as the basic reproductive number represents the average number of newly infected cells per infected cell at the beginning of infection.

# 2 Details of the hybrid method

## 2.1 Description of algorithm

At any given time, let $\mathbf{V}_l$ and $\mathbf{V}_s$ be vectors containing the large and small cell populations. We can then define the reduced system $d\mathbf{V}_l/dt = \mathbf{F}_l(\mathbf{V}_l)$ derived from the full system by: (1) retaining only the equations for the large cell populations $\mathbf{V}_l$; (2) keeping constant the contributions of the small populations $\mathbf{V}_s$. If the components of $\mathbf{V}_l$ are sufficiently large, there will be a time interval $(t, t+\tau)$, where the deterministic solution of the reduced ODE will approximate the trajectories of the large populations in a stochastic implementation of the full system.

When an infection event occurs (either free virus transmission or cell-to-cell transmission), the target cell moves from its current subpopulation to its new subpopulation of higher multiplicity. We refer to the original cell population as the *minus cell subpopulation* (because it has lost a member), and to the new subpopulation as the *plus cell subpopulation* (because it has gained a member).

The events in the model are uninfected cell generation, cell death, and infection with up to $S$ copies of combinations of the $2^k$ viral strains. In Gillespie's method, every event $\nu$ has a given propensity $a_\nu(\mathbf{V})$. The time at which the next event $\nu$ will occur is exponentially distributed with intensity $a_\nu(\mathbf{V})$. In the hybrid approach, uninfected cell generation, death of large populations, and infection where both the minus cell population and plus cell population are large are modeled deterministically (using the reduced system), while cell death and infection of small populations are modeled stochastically, with propensities $a_\nu(\mathbf{V}_s, \mathbf{V}_l(t))$ that now vary continuously with time.

If the minus cell population and the plus cell population are of different size classification (one is small and the other is large), then infection between these two populations is modeled with a "half and half reaction" (see next Section). For further details on the hybrid algorithm, see [8].

## 2.2 Classification of reactions

This is an explanation of how individual reactions are designated small or large. This is a system with $N = 4$, no mutation, and only free virus transmission. Let $Z(t) = \sum_{i=1}^{4} x_i(t)$ be the sum of all infected subpopulations. The equations in the absence of synaptic transmission are

$$\begin{aligned}
\dot{x}_0 &= \lambda - \beta Z x_0 - d x_0 && (10)\\
\dot{x}_1 &= \beta Z x_0 \boldsymbol{- \beta Z x_1} - a x_1 && (11)\\
\dot{x}_2 &= \boldsymbol{\beta Z x_1} - \beta Z x_2 - a x_2 && (12)\\
\dot{x}_3 &= \beta Z (x_2 - x_3) - a x_3 && (13)\\
\dot{x}_4 &= \beta Z (x_3) - a x_4 && (14)
\end{aligned}$$

Assume we are at time $t^*$ and we have size threshold $\mathcal{M}^*$. Assume that that $x_0(t^*) \geq \mathcal{M}^*$, $x_1(t^*) \geq \mathcal{M}^*$, $x_2(t^*) < \mathcal{M}^*$, $x_3(t^*) < \mathcal{M}^*$, and $x_4(t^*) < \mathcal{M}^*$. Then at time $t^*$ we have that populations $x_0$ and $x_1$ are large and all other populations are small.

Since $x_0$ and $x_1$ are large and all other populations are small, we only update Equations (10) and (11) deterministically. The reactions that are included in Equations (12)-(14) are treated stochastically as a continuous time Markov chain (see the jump and rate table below), and these differential equations are not updated deterministically. The stochastic reactions are given by

| Jump | Rate |
|---|---|
| $x_2 \to x_2 + 1$ | $\beta Z x_1$ |
| $x_2 \to x_2 - 1$ | $a x_2$ |
| $x_2 \to x_2 - 1,\ x_3 \to x_3 + 1$ | $\beta Z x_2$ |
| $x_3 \to x_3 - 1$ | $a x_3$ |
| $x_3 \to x_3 - 1,\ x_4 \to x_4 + 1$ | $\beta Z x_3$ |
| $x_4 \to x_4 - 1$ | $a x_4$ |

where the first reaction listed is a "half and half" reaction (see paragraph below). We note that this definition of the system only holds for time $t^*$, as at each new time-step, we reclassify which populations are large and small (based on size threshold $\mathcal{M}$), and so the designation as "large" or "small" will dynamically change in the course of a simulation for each population and their associated reactions.

The reaction that infects population $x_1$ with rate $\beta Z x_1$ is updated deterministically in Equation (11) and also is included as a stochastic reaction in the Markov chain model because population $x_2$ is small. Therefore, we lose a singly infected cell both deterministically and stochastically, and only gain an $x_2$ stochastically. Therefore, we designate the reaction that infects populations $x_1$ a "half and half" reaction. This means that $x_1$ is updated deterministically in Equation (11) and also stochastically because population $x_2$ is small, but during the stochastic reaction we only gain an $x_2$ and do not lose an $x_1$ (see the first reaction in the table above). So in essence we lose $x_1$ to infection deterministically, and gain $x_2$ by infection stochastically (but do not lose an $x_1$). More generally, we have a half and half reaction whenever the minus population is large and the plus population is small, or the minus population is small and the plus population is large.

In this system, since there is only free virus transmission the plus population is always $x_{i+1}$. However, in a system with synaptic cell-to-cell transmission and a mutant strain, the plus population can be $x_{i+1,j}$, $x_{i,j+1}$, $x_{i+S,j}$, $x_{i,j+S}$, etc. So under these assumptions, in the ODE system given by Equations (10)-(14), we have that all unbolded terms in Equations (10)-(11) are the large deterministic reactions, the **bolded** terms are the half and half reactions, and all other terms are the stochastic reactions.

### 2.3 Choosing a size threshold $\mathcal{M}$

The rate of stochastic extinction of the hybrid method should match the rate of stochastic extinction of the simulations in the fully stochastic case [9, 10]. As described in the main text, we define $\hat{\mathcal{M}}$ as the lower bound on the size threshold $\mathcal{M}$. Therefore we have that

$$\hat{\mathcal{M}} = \lceil \ln\left(1 + \frac{R_0 - 1}{\delta R_0}\right) / \ln R_0 \rceil, \tag{15}$$

where $\lceil . \rceil$ denotes the ceiling function. Since increasing $R_0$ monotonically leads to a higher rate of successful infections, we see that increasing $R_0$ leads to decreasing $\hat{\mathcal{M}}$. Table B shows lower bound $\hat{\mathcal{M}}$ for a variety of $R_0 > 1$ and difference thresholds $\delta$.

When implementing the hybrid algorithm, the ODE step size $h$ must also be taken into account. The step size $h$ must be small enough such that i) in the completely deterministic case it gives an accurate numerical solution of the ODE solution, and ii) in the hybrid case the stochastic events and dynamics are captured. This only becomes an issue when size threshold $\mathcal{M}$ is very large (so there are many populations and $\tau$ becomes quite small for multiple reactions) but there are both small and large populations (so that some populations are still updating deterministically). In this case, the ODEs for the uninfected population becomes stiff and smaller step size $h$ might be needed to capture the initial decline of the uninfected cell population.

| $R_0$ | $\hat{\mathcal{M}}$ for $\delta = 10^{-4}$ | $\hat{\mathcal{M}}$ for $\delta = 10^{-5}$ | $\hat{\mathcal{M}}$ for $\delta = 10^{-6}$ |
|---|---|---|---|
| 1.001 | 2399 | 4617 | 6912 |
| 1.01 | 463 | 694 | 925 |
| 1.1 | 72 | 96 | 120 |
| 1.5 | 21 | 26 | 32 |
| 2 | 13 | 16 | 19 |
| 4 | 7 | 9 | 10 |
| 6 | 6 | 7 | 8 |
| 8 | 5 | 6 | 7 |

Table B : $\hat{\mathcal{M}}$ values for a variety of $R_0 > 1$ and difference thresholds $\delta$. We define $\hat{\mathcal{M}}$ according to Equation (15).

### 2.4 Comparison with tau-leaping method

The tau-leaping method/adaptive tau-leaping method measures how many times a given reaction "fires" in a given time interval $[t, t + \tau)$. Quantity $\tau$ is the step size (either constant or variable in the adaptive framework) which is usually determined as the largest value such that the leap condition is satisfied, which requires that no reaction propensity function changes by a "significant" amount in the interval $[t, t + \tau)$. If the number of "firings" of each reaction in the interval $[t, t + \tau)$ is much greater than 1, then the adaptive tau-leaping method will result in a significantly faster

stochastic simulation (compared to the basic Gillespie algorithm, which performs each reaction sequentially). Without fully implementing the tau-leaping method, it is possible to show that it is significantly slower than the hybrid method used in our paper. The argument consists of two parts.

(A) We first show that updating the system at each step is less computationally expensive for the hybrid method than for the tau-leaping method. Comparing a stochastic update to a deterministic step using the differential equations for our model, it is straightforward to see from the program code that the deterministic step involves fewer floating point operations compared to a stochastic update. One contributing factor for this is the number of reactions (which is quite large), and that the stochastic updates treat all of these reactions individually. In addition to fewer operations, the deterministic step does not require the generation of any random numbers (which is computationally expensive), whereas the stochastic update step (either basic Gillespie or tau-leaping method) requires a random number to be generated for each reaction channel for each time interval. Therefore, if for instance the hybrid method and tau-leaping method used the same step sizes (i.e. the system gets updated at exactly the same time points $t = 0, t_1, t_2, t_3, \ldots$) for both methods, then the hybrid method will run faster because it involves less operations (and less expensive operations such as pseudo-random number generation) at each step.

The tau-leaping method is also much more operationally expensive (again if updated at the same time points) than just the basic Gillespie method, because of the additional operations needed to calculate $\tau$ and confirm it satisfies the leap condition.

Next, we show that the step sizes for tau-leaping will not be larger than the steps for hybrid:

(B) Without fully coding the tau-leaping method, we can calculate if different values of $\tau$ satisfy the leap condition. One such method is Equation (3) from [11], which is given by

$$|a_j(\mathbf{x} + \mathbf{\Lambda}(\tau; \mathbf{x}) - a_j(\mathbf{x})| \leq \epsilon a_0(\mathbf{x}), \quad \text{for all } j = 1, 2 \ldots, M. \tag{16}$$

Here, $M$ is the number of reactions, $0 < \epsilon \leq 1$ is a pre-specified error control parameter, $\mathbf{x}(t) = (x_0(t), x_1(t), \ldots, x_N(t))$ is the state vector of all subpopulations at time $t$ (here we assume only one strain for simplicity of writing out the vector), $a_j$ is the propensity of the $j$th reaction (where reactions are uninfected cell generation, cell death, and infection of target cells of different subpopulations), $a_0$ is the sum of all the propensity functions, and $\mathbf{\Lambda}(\tau; \mathbf{x}) = \mathbf{x}(t + \tau) - \mathbf{x}(t)$ is the change in the state vector of the system over the interval $[t, t + \tau)$. Another method is Equation (17) from [12], which is given by

$$\Delta_\tau a_j(\mathbf{x}) \leq \max\{\epsilon a_j(\mathbf{x}), c_j\}, \quad j = 1, 2, \ldots, M. \tag{17}$$

Here, $\Delta_\tau a_j(\mathbf{x})$ is the change in propensity function $a_j$ from time $t$ to time $t + \tau$, and $c_j$ is the minimum positive change by which reaction $a_j(\mathbf{x})$ can change (which is included as if $a_j(\mathbf{x})$ approaches zero then $\tau$ could be forced to approach zero as well). We set $\epsilon = 0.1$. In order to demonstrate that the maximum $\tau$ allowed by the leaping condition is smaller than the deterministic step size (denoted by $h$), we find that using $\tau = h$ violates both Equations (16) and (17) at all arbitrarily tested points we used ($t = 0.001, 0.01, 1, 2, 5$). This implies that $\tau$ must be smaller than the deterministic step size, that is, hybrid steps are larger (in simulated infection time) than tau-leaping steps.

To summarize: (A) above shows that hybrid steps are faster (in computer time) than tau-leaping steps, (B) shows that hybrid steps are larger than tau-leaping steps (in simulated infection time), so (A) and (B) together imply that the hybrid method is faster (less computer time to get to larger simulated infection time) than tau-leaping.

Next, we argue that the tau-leaping method is not a good choice for our system. The leap condition is best satisfied if all of the reaction propensity functions are large [13]. However, in our system, as described above, we have the continued persistence of both small and large populations that interact with each other. We begin with $10^9$ uninfected cells and a single infected cell. In the timespan under consideration, the number of infected cells with grow to the order of $10^8$ cells, however, the total number of infected cells will be subdivided into many subpopulations of infected cells, and we will always have many small populations ($< 10$) if $k$ and $N$ are sufficiently large. Therefore, measuring how many times a given reaction "fires" in a specified time interval when it affects populations that are of the order of $10^0$ cells and other populations of $10^9$ cells does not make much sense. This is because small fluctuations in small populations can have a large effect on the evolutionary dynamics of the infection, but small fluctuations in sufficiently large populations (such as uninfected or highly prevalent cells that are singly infected with the wild type virus) often have very little effect on the dynamics.

Instead, multi-scale methods make more sense for systems with populations of vastly different scales, and so we implement our hybrid method where large populations are treated deterministically (which is both more computationally efficient and makes sense because small fluctuations in large populations have little effect) and small populations are treated stochastically (because small fluctuations in small populations can have large effects which are not captured by deterministic models).

In conclusion, the tau-leaping method is not an optimal choice for our system, and it is also clearly slower than our hybrid method. For these reasons, we did not implement it fully in this study, but instead, provided a comparison with the conventional Gillespie method (see Table 2 of the main text).

### 2.5 Comparison with piecewise deterministic Markov processes

Our stochastic-deterministic algorithm is similar to piecewise deterministic Markov processes (PDMPs) [14, 15, 16], in which a deterministic motion/process is modified to include discrete stochastic Markov jumps. PDMPs have been used to model a wide variety of biological systems including processes in population dynamics [17, 18], genetics [19, 20, 21], and physical chemistry [22, 23]. There has also been much work on the efficient numerical implementation of PDMPs (including in [24, 16, 25, 26]), as simulations can be computationally expensive and difficult. In particular, [26] proposes a simulation algorithm for the stochastic Hodgkin-Huxley model that approximates the exact solution by fitting the log-survival function of the inter-jump dwell time with a piecewise linear one, and in [24] a thinning algorithm that depends on the jump rate bound is developed to efficiently simulate similar Hodgkin–Huxley type models.

Our algorithm is different from standard PDMPs because the different reactions in the system bounce back and forth from deterministic to stochastic implementations based on the size of the populations involved in the reactions and the size threshold $\mathcal{M}$. While simulation methods from PDMPs are not directly applicable to our algorithm, both theoretical analysis of PDMPs and methods to efficiently implement them are likely important for future work in virus dynamics and hybrid stochastic-deterministic algorithms.

## 3 Comparison of the ODE and stochastic/hybrid simulations in the context of infection dynamics

The deterministic ODEs do not provide information on the distribution of mutant numbers. The hybrid method is especially useful here, because it allows for efficient stochastic computations at

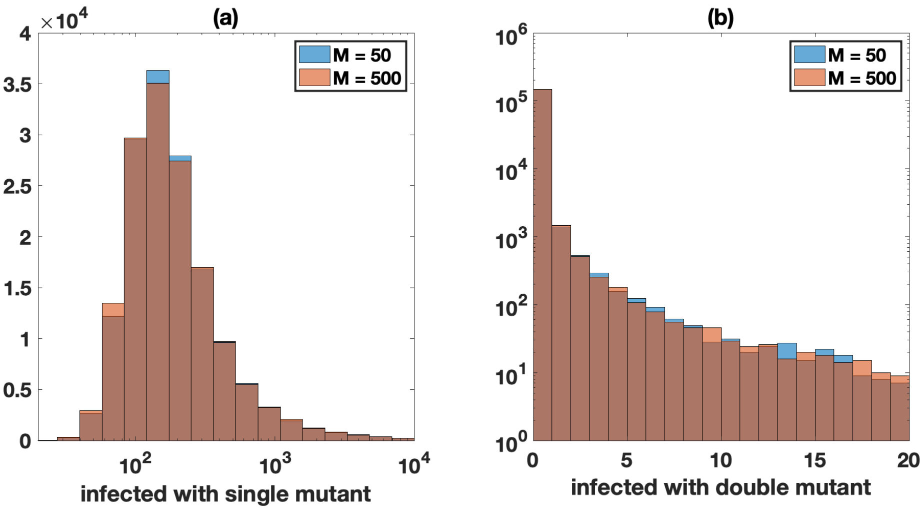


Fig S3: Histograms of the number of cells infected with the neutral single and double mutant strain of the virus (double mutant model, $F = 1$ for all strains) when the infected cell population reaches $10^6$ cells for hybrid simulations with size threshold $\mathcal{M} = 50$ and $\mathcal{M} = 500$. Simulations include only free virus transmission. For each size threshold $1.5 \times 10^5$ simulations were performed. A Number of cells infected with one of the single mutant strains. B Number of cells infected with the double mutant strain. The number of cells infected with the double mutant is not statistically different for $\mathcal{M} = 50$ and $\mathcal{M} = 500$ ($p > 0.1$ by Kolmogorov-Smirnov test). The parameters are $N = 3,\ \mu = 3 \times 10^{-5},\ \lambda = 1.59 \times 10^7,\ \beta = 3.60 \times 10^{-9},\ \gamma = 0,\ a = 0.45,\ d = 0.016$, and $R_0 = 8$.

large population sizes, and can generate mutant distributions rather than just averages.

Fig S3 shows an example of histograms of the number of cells infected with a neutral single and double mutant strain of the virus when the infected cell population reaches $10^6$ cells for hybrid simulations with size threshold $\mathcal{M} = 50$ and $\mathcal{M} = 500$ and only free virus transmission. Here, we see that for $R_0 = 8$, size threshold $\mathcal{M} = 50$ is large enough to provide a good approximation of the fully stochastic dynamics. Fig S4 shows that for $R_0 = 1.5$, $\mathcal{M} = 50$ is not large enough to capture the stochastic dynamics, and larger size thresholds are needed. As discussed in the main text, lower $R_0$ is associated with larger stochastic effects.

Furthermore, an important benchmark of infection is the emergence of mutant strains of the virus. The hybrid method allows us to compute full distributions for when the first mutant is expected to appear stochastically (by a mutation from a wild-type virus). Fig S5 demonstrates how the deterministic model predictions for the first mutant virus to appear can differ from averages over many stochastic runs simulated using the computationally efficient hybrid algorithm.

Finally, while we have shown that ODEs cannot accurately describe the average behavior of the stochastic model, the hybrid method (with a sufficient size threshold) is able to do so. Fig S6A compares the number of cells infected with the mutant at infected population size $10^4$ and Fig S6B compares the time at first mutant generation for simulations in which all infected cell populations are always treated stochastically versus simulations with $\mathcal{M} = 50$. In both cases, the Kolmogorov-Smirnov test between the two distributions does not suggest that there is a statistically significant difference.

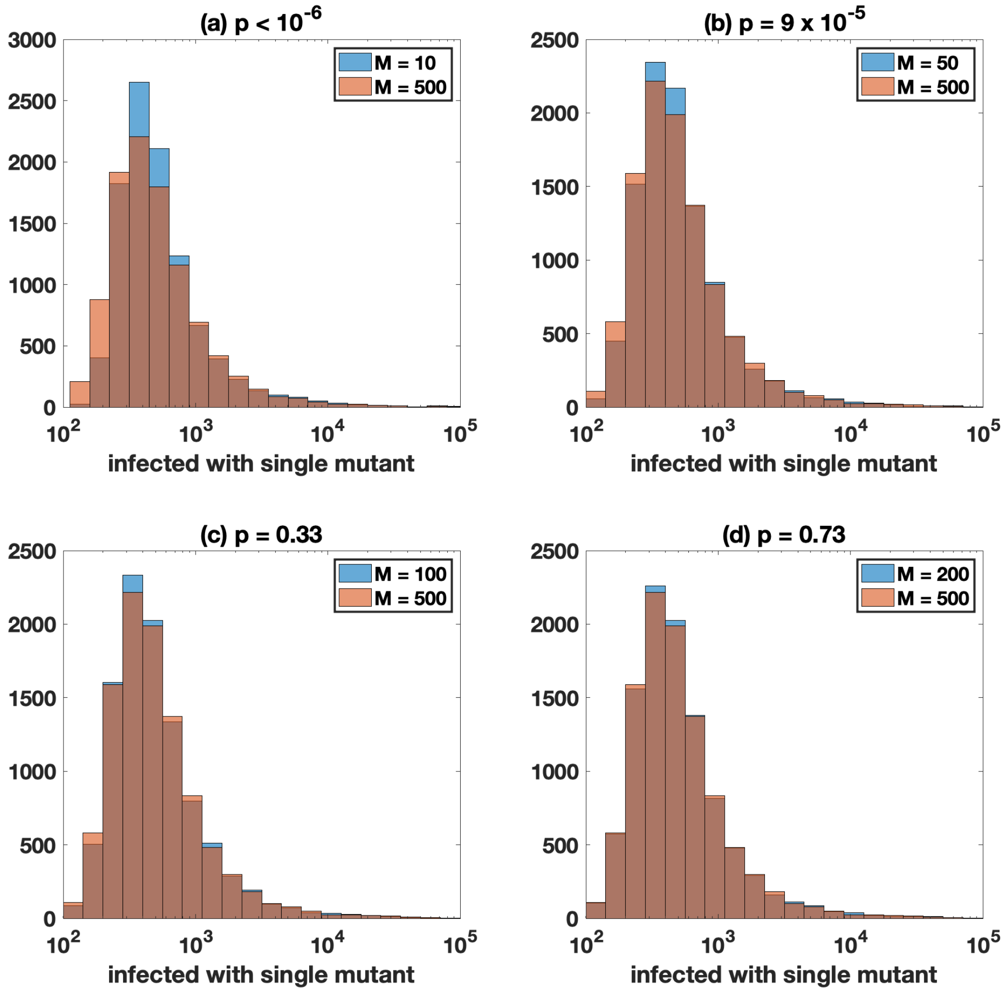


Fig S4: Histograms of the number of cells infected with the neutral single mutant strain of the virus (single mutant model, $F_{\text{wild-type}} = F_{\text{mutant}} = 1$) when the infected cell population reaches $10^6$ cells for hybrid simulations with only free virus transmission. Histograms for each size threshold represent $10^4$ simulations. The $p$-value from Kolmogorov-Smirnov test is shown for each comparison. A Size threshold $\mathcal{M} = 10$ and $\mathcal{M} = 500$. B Size threshold $\mathcal{M} = 50$ and $\mathcal{M} = 500$. C Size threshold $\mathcal{M} = 100$ and $\mathcal{M} = 500$. D Size threshold $\mathcal{M} = 200$ and $\mathcal{M} = 500$. Here $N = 1$ and the other parameters are $\mu = 3 \times 10^{-5},\ \lambda = 1.59 \times 10^7,\ \beta = 3.60 \times 10^{-9},\ \gamma = 0,\ d = 0.016$, and $R_0 = 1.5$.

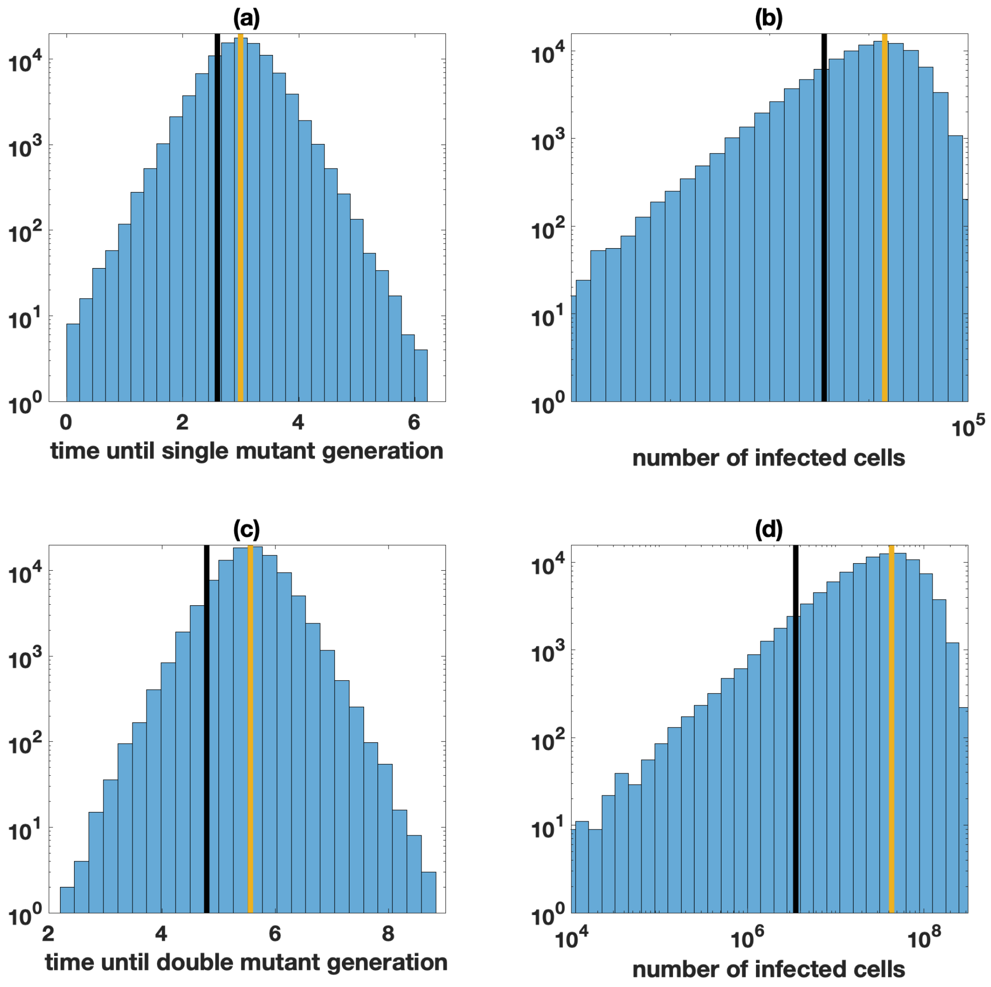


Fig S5: Histograms of generation time of first single/double mutant virus in the double mutation model in the context of only free virus transmission (with all strains neutral). Simulations in which infections are not established are discarded when calculating the averages. The deterministic prediction is denoted with the black vertical line and the hybrid average is denoted with the yellow vertical line. Histograms represent $10^5$ hybrid simulations with size threshold $\mathcal{M} = 50$. A Time until either single mutant generation. The deterministic prediction that the first single mutant virus (of both strains) will be generated is around 2.6 days, whereas in the stochastic case it is around 3 days. B Number of infected cells at first single mutant generation. The deterministic prediction is that the number of infected cells is around $3.5 \times 10^3$, whereas in the stochastic case it is around $1.4 \times 10^4$. C Time until double mutant generation. The deterministic prediction that the first double mutant virus will be generated is around 4.8 days, whereas in the stochastic case it is around 5.6 days. D Number of infected cells at first double mutant generation. The deterministic prediction is that the number of infected cells is around $3.5 \times 10^6$, whereas in the stochastic case it is around $4.3 \times 10^7$. The parameters are $N = 3$, $\mu = 3 \times 10^{-5}$, $\lambda = 1.59 \times 10^7$, $\beta = 3.60 \times 10^{-9}$, $\gamma = 0$, $a = 0.45$, $d = 0.016$, and $R_0 = 8$.

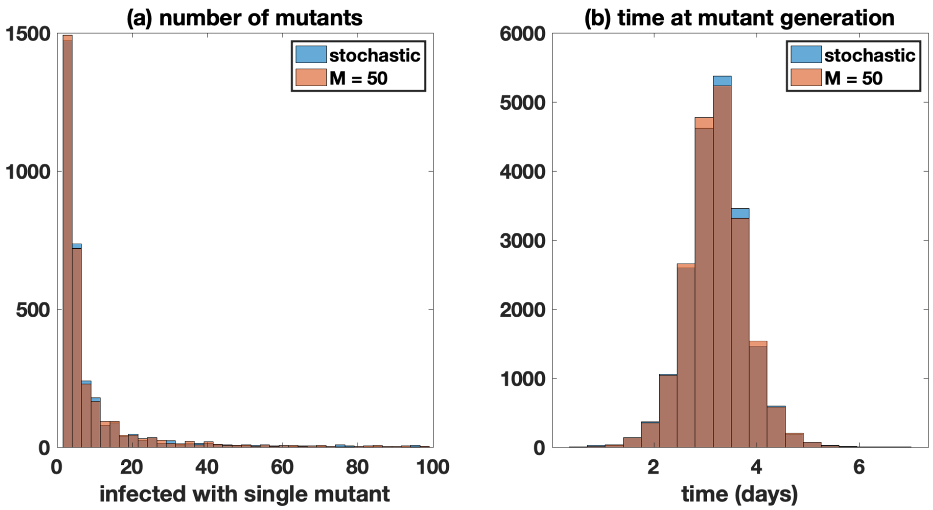


Fig S6: Comparing hybrid simulations with $\mathcal{M} = 50$ (red) with simulations in which the infected subpopulations are always treated stochastically (blue). Simulations in which infections are not established are discarded when calculating the averages. Histograms represent $2 \times 10^4$ simulations. A The number of cells infected with the mutant at infected population size $10^4$. B The time at first mutant generation. The parameters are $F_{\text{wild-type}} = F_{\text{mutant}} = 1,\ N = 3,\ \mu = 3 \times 10^{-5},\ \lambda = 1.59 \times 10^7,\ \beta = 3.60 \times 10^{-9},\ \gamma = 0,\ a = 0.45,\ d = 0.016$, and $R_0 = 8$.

# 4 Impact of multiple infection and synaptic transmission on mutant evolution: additional results

We consider the effect of multiple infection on mutant dynamics, as well as different scenarios where fitness is dependent upon the contents of a cell (i.e. complementation, interference). Thus, the multiplicity of infection is important. Widely varying estimates for average infection multiplicities have been published [27, 28, 29, 30], and there is some uncertainty. Fig S7 shows the deterministic time series for an infection with a single neutral mutant strain in the presence of only free virus or only synaptic transmission. For only free virus transmission, the multiplicity of infection near peak infection is 2 and reaches a maximum of 6 just after peak infection. For only synaptic transmission, the multiplicity of infection near peak infection is 7 and reaches a maximum of 17 just after peak infection. Stochastic simulations give similar values for the multiplicity of infection, and are very consistent with standard deviations usually less than $10^{-2}$. Therefore, as we increase the contribution of synaptic transmission, the number of coinfected cells increases, which leads to more pronounced effects of interactions between different virus strains within cells such as complementation and interference.

We can also use the hybrid method to determine the distribution of multiplicity of infection near peak infection, based on different transmission strategies. Fig S8 shows histograms of the number of cells infected with different numbers of viral copies on average, for only free virus transmission (Fig S8A) and half free virus transmission and half synaptic transmission (Fig S8B). The distribution for only synaptic transmission will be the same as only free virus transmission, except the number of viral copies (horizontal axis) is scaled by $S$ (i.e. $1 \to S,\ 2 \to 2S$) and all other values will contain no cells. As done in [31], the steady states for each population can be determined analytically.

Mutant evolution is impacted by multiple infection [32]. Fig S9 shows histograms of cells infected with neutral single and double mutants in the presence and absence of multiple infection at relatively low virus loads. Here, the average number of mutants is the same, whether multiple infection is assumed to occur or not because of the low multiplicity of infection at lower virus loads. This figure should be compared with Fig 2 of the main text where the mutant numbers were mea-

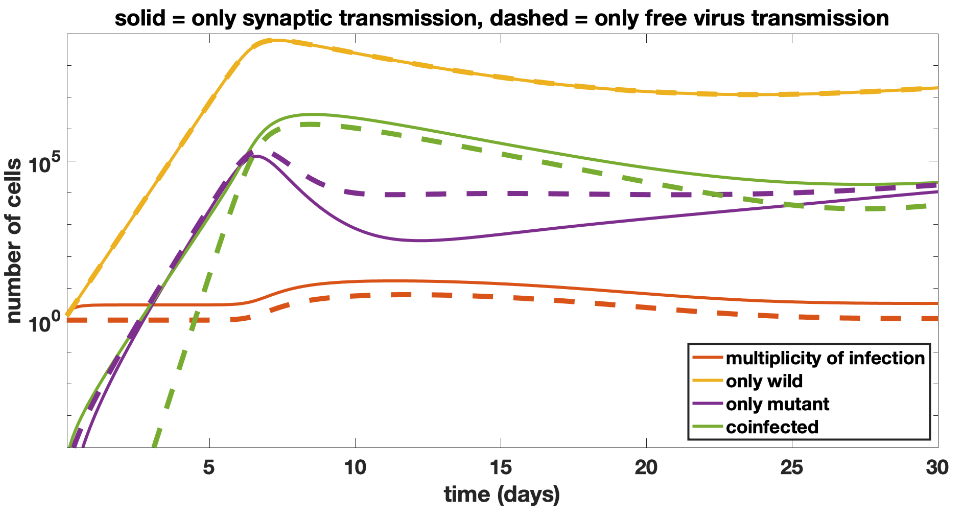


Fig S7: Deterministic time series evolution of an infection with a neutral mutant strain with only free virus (dashed lines, $\gamma = 0$) or only synaptic transmission (solid lines, $\beta = 0$). Parameters are $N = 25,\ \beta + \gamma = c = 3.6 \times 10^{-9},\ \mu = 3 \times 10^{-5},\ \lambda = 1.59 \times 10^7,\ a = 0.45$, and $d = 0.016$. The multiplicity of infection is shown with the red lines, the number of cells infected with only the wild-type virus are shown with the yellow lines, the number of cells infected with only the mutant are shown with the purple lines, and the number of cells coinfected with both the wild-type and mutant are shown with the green lines.

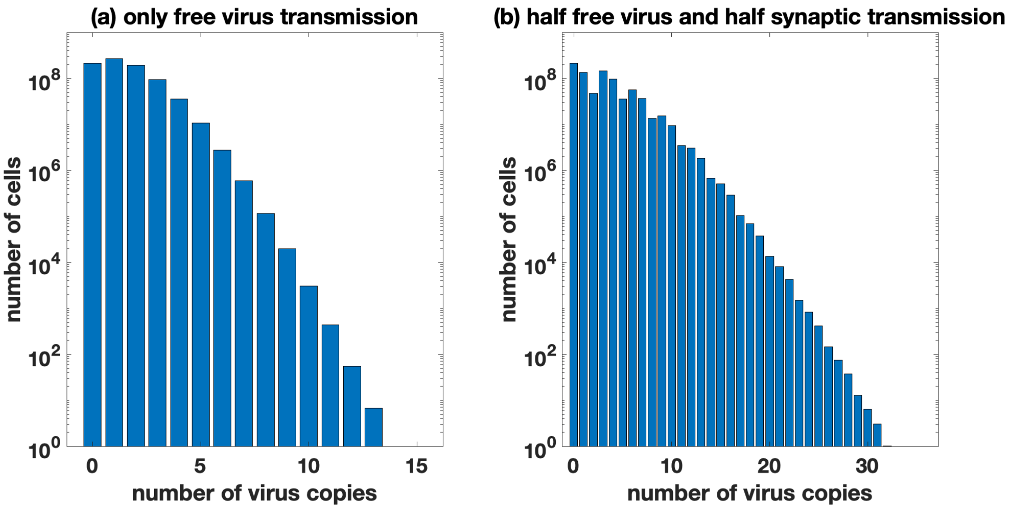


Fig S8: Histograms of the multiplicity of infection near peak infection. A Histograms for the average number of cells infected with the given number of viral copies for only free virus transmission. B Histograms for the average number of cells infected with the given number of viral copies for half free virus transmission and half synaptic transmission. The horizontal axis is the number of virus copies and the vertical axis is the average number of cells that are infected with that number of viral copies near peak infection. Infected with zero copies corresponds to the uninfected cells. Histograms were averaged over $5 \times 10^2$ hybrid simulations with size threshold $\mathcal{M} = 50$. Simulations are stopped when the infected cell population is close to peak infection ($6 \times 10^8$ cells). Parameters are $\beta + \gamma = c = 3.6 \times 10^{-9},\ \mu = 3 \times 10^{-5},\ \lambda = 1.59 \times 10^7,\ a = 0.45$, and $d = 0.016$, and maximum multiplicity of infection $N$ is set to be large enough such that no cells reach this threshold.

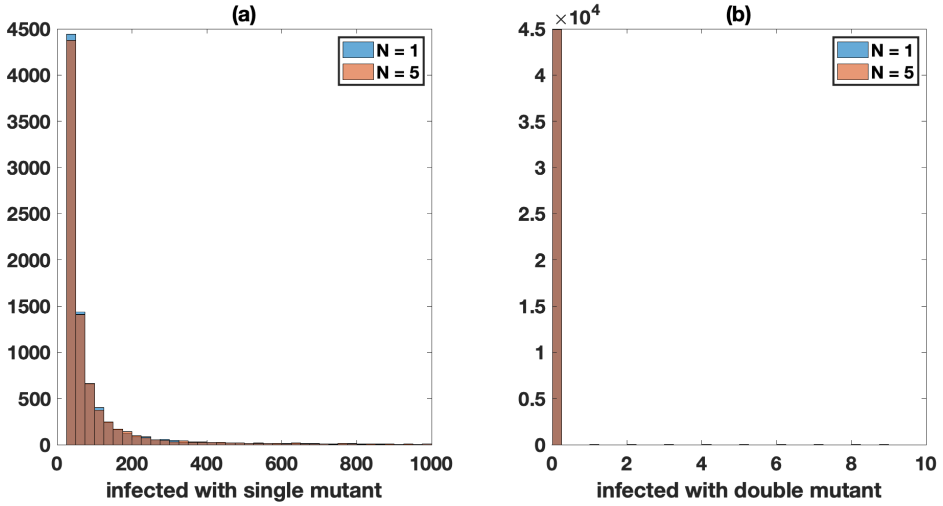


Fig S9: Neutral mutant evolution in the absence of synaptic transmission, comparing simulations with single infection only ($N = 1$, blue) and in the presence of multiple infection ($N = 5$, red). For both panels, the Kolmogorov-Smirnov test between the two distributions suggests that they are not statistically different. A Number of cells infected with one of the single mutant strains. B Number of cells infected with the double mutant strain. Distributions represent $4.5 \times 10^4$ hybrid simulations with size threshold $\mathcal{M} = 50$. Simulations are stopped at a low viral load ($10^5$ cells). The other parameters are as in main text Fig 2 ($F_{\text{wild-type}} = 1,\ F_{\text{mutant}} = 1,\ \mu = 3 \times 10^{-5},\ \lambda = 1.59 \times 10^7,\ \beta = 3.60 \times 10^{-9},\ \gamma = 0,\ a = 0.45$, and $d = 0.016$).

sured at a higher population size, and a significant increase in the number of mutants was observed in the presence of multiple infection.

Fig S10 is similar to Fig 2 of the main text, but it studies the numbers of cells infected with disadvantageous (advantageous) mutants with and without multiple infection, in the presence of only free virus transmission. As in the main text Fig 2, the number of cells was recorded near peak infection, and the maximum multiplicity parameter was $N = 1$ for simulations with single infection only, and $N = 11$ for the model including multiple infection. For non-neutral mutants we observe the same trends discussed in the main text, that is multiple infection results in an increased number of cells infected with the mutant as well as increased variation in the number of mutants. In the case of a disadvantageous single mutant with a 10% disadvantage, we observe a 2.1-fold increase in the mean number of cells infected with the mutant, and a 3.3-fold increase in the standard deviation when multiple infection is included. In the case of an advantageous single mutant with a 10% advantage, we observe a 1.9-fold increase in the mean number of cells infected with the mutant, and a 1.5-fold increase in the standard deviation when multiple infection is included.

Multiple infection also increases the prevalence of neutral triple mutants. Fig S11 compares the number of triple mutants with and without multiple infection near peak infection, again for only free virus transmission. The number of triple mutants is small, but the probability to have at least one cell infected by a triple-mutant is much higher when multiple infection is allowed (3.3% under multiple infection, which is about 1.7 times higher than the 1.9% under single infection).

With multiple infection, we can also include synaptic transmission. During synaptic cell-to-cell transmission, if the fitness of all strains is 1, we assume that $S \geq 1$ viruses are always transferred to the target cell per infection event. During an infection event, upon selection and infection of each virus, the chosen viral copy has a probability to mutate. This greatly increases the ability of synaptic transmission to generate mutant strains of virus. To see this, consider infection from a cell that is infected only by the wild-type. During a free virus transmission event, there is only one

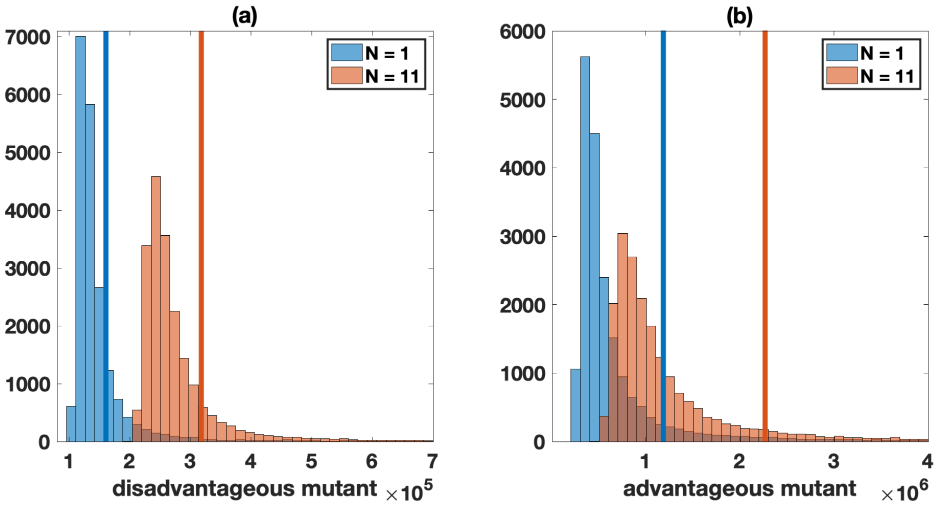


Fig S10: Non-neutral (disadvantageous and advantageous) mutant evolution in the absence of synaptic transmission, comparing simulations with single infection only ($N = 1$, blue) and in the presence of multiple infection ($N = 11$, red). The mean values are shown by the vertical lines (blue for single infection only and red for multiple infection). For both panels, the Kolmogorov-Smirnov test between the two distributions gives a $p$-value less than $10^{-6}$. A Disadvantageous mutant; here $F_{\text{mutant}} = 0.81$. The average under single infection only is approximately $1.6 \times 10^5$ and for multiple infection is approximately $3.2 \times 10^5$. B Advantageous mutant with interference; here $F_{\text{mutant}} = 0.99$. The average for single infection is approximately $1.2 \times 10^6$ and for multiple infection is approximately $2.3 \times 10^6$. Histograms represent $2 \times 10^4$ hybrid simulations with size threshold $\mathcal{M} = 50$. Simulations are stopped when the infected cell population is close to peak infection ($6 \times 10^8$ cells). The other parameters are $F_{\text{wild-type}} = 0.9$, $\mu = 3 \times 10^{-5}$, $\lambda = 1.59 \times 10^7$, $\beta = 4 \times 10^{-9}$, $\gamma = 0$, $a = 0.45$, and $d = 0.016$.

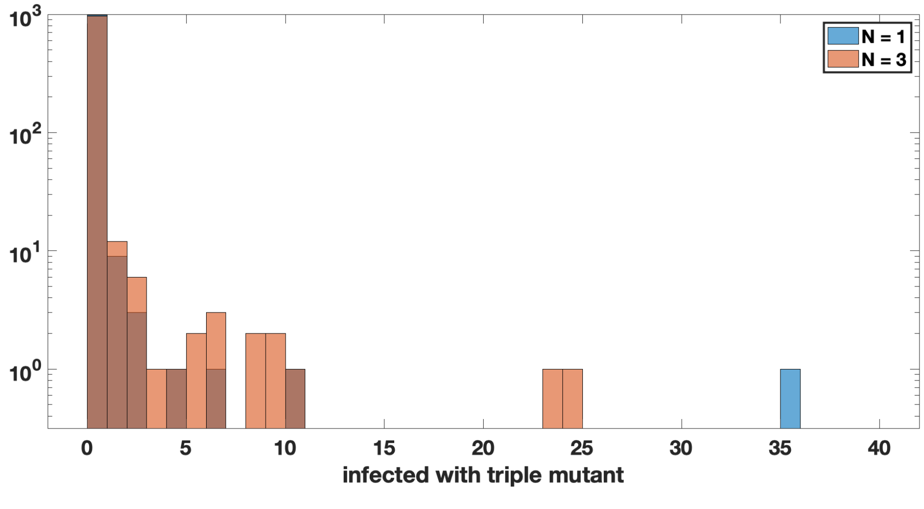


Fig S11: Presence of the triple mutant strain in the absence of synaptic transmission, comparing simulations with single infection only ($N = 1$, blue) and in the presence of multiple infection ($N = 3$, red). The probability to have at least one cell infected by a triple-mutant is 3.3% under multiple infection, which is about 1.7 times higher than that under single infection (1.9%). This result is significant with $p = 2.5 \times 10^{-3}$ by the Z-test, with $2.7 \times 10^4$ runs under single infection and $1.9 \times 10^3$ runs under multiple infection. Histograms represent $10^3$ hybrid simulations with size threshold $\mathcal{M} = 50$. Simulations are stopped when the infected cell population is close to peak infection ($6 \times 10^8$ cells). All strains are neutral ($F = 1$) and all other parameters are $\mu = 3 \times 10^{-5}$, $\lambda = 1.59 \times 10^7$, $\beta = 3.60 \times 10^{-9}$, $\gamma = 0$, $a = 0.45$, and $d = 0.016$.

chance for mutation into a mutant strain. However, during a synaptic transmission event, there are $S$ chances for mutation into a mutant strain.

If the fitness $F$ of any strain is less than 1, then there is also a chance that an unsuccessful infection event occurs. In particular, the probability of a completely unsuccessful infection event in which no viral copies are transferred during a free virus transmission event is $1 - F$, and in the case of synaptic transmission it is $(1 - F)^S$. Therefore, the rate at which an infection grows is influenced by the relative contribution of free virus versus synaptic transmission (and increases monotonically with increasing contribution of synaptic transmission).

Including synaptic transmission results in increased multiplicity of infection. Therefore, we need to adjust the maximum infection multiplicity used in the model, to avoid spurious accumulation of cell numbers at the end of the infection cascade. For our model, for $R_0 = 8$, $S = 3$, and 100% synaptic transmission, most cells do not become infected with more than 26 copies of virus by the time of peak infection. Therefore, we set $N = 25$ for all simulations that include synaptic transmission (see also Section 1.3).

Fig S12 shows the number and histograms of cells infected with a neutral mutant ($F_{\text{wild-type}} = F_{\text{mutant}} = 1$, so infection always goes through) near peak infection for multiple combinations of free virus and synaptic transmission. As synaptic transmission is better at generating mutants, larger contributions of synaptic transmission result in larger mutant numbers and the number of mutants decreases monotonically with increasing contribution of free virus transmission.

Fig S13 should be compared with Fig 3 of the main text. For these simulations, in order to explicitly analyze the effect of multiple infection and coinfection, we initially begin with a single infected cell, which is coinfected with a single copy of both the wild-type and mutant virus. Further, we turn off mutation (set $\mu = 0$), so that synaptic transmission no longer results in a higher rate of mutant generation. Fig 3 of the main text studies disadvantageous (zero fitness) mutant spread in the absence of mutations, with and without complementation. Fig S13 considers advantageous mutants with and without interference. It shows histograms of cells infected with a 10% advantageous mutant with and without interference for either 100% free virus or 100% synaptic transmission.

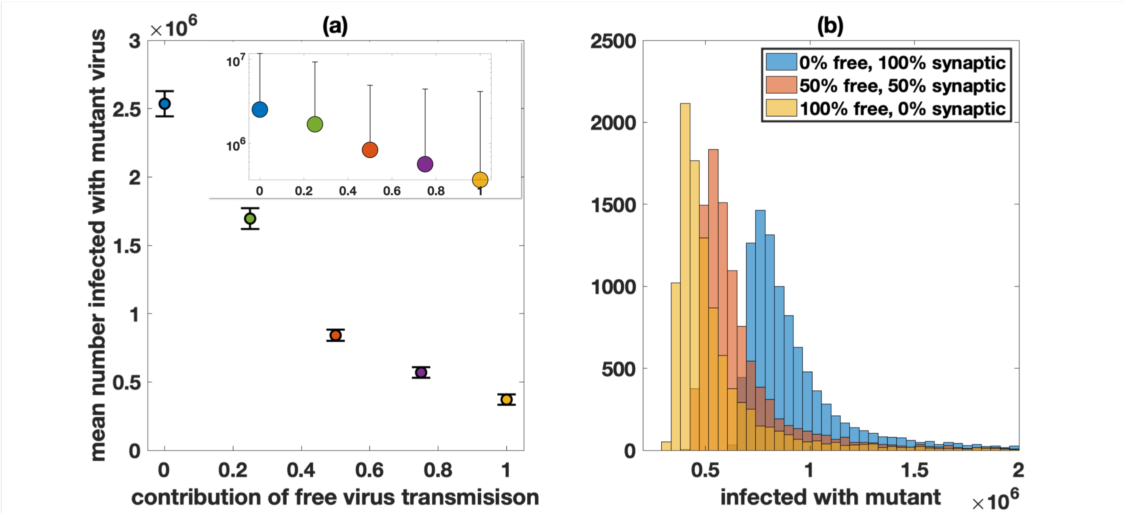


Fig S12: Neutral mutant evolution in the presence of synaptic transmission, comparing simulations with different combinations of free virus and synaptic transmission (single mutation only). A Number of cells infected with the mutant strain under different transmission strategies. The horizontal axis is the percent contribution of free virus transmission. Standard error bars are shown in the main figure, and standard deviation bars are shown in the inset. B Histograms for the number of cells infected with a single mutant for the different strategies, representing $10^4$ hybrid simulations with size threshold $\mathcal{M} = 50$. Simulations are stopped when the infected cell population is close to peak infection ($6 \times 10^8$ cells) and simulations where no infection is established are thrown out. Here $F_{\text{wild-type}} = 1,\ F_{\text{mutant}} = 1,\ S = 3,\ N = 25,\ \beta + \gamma = c = 3.6 \times 10^{-9}$, and the other parameters are $\mu = 3 \times 10^{-5},\ \lambda = 1.59 \times 10^7,\ a = 0.45$, and $d = 0.016$.

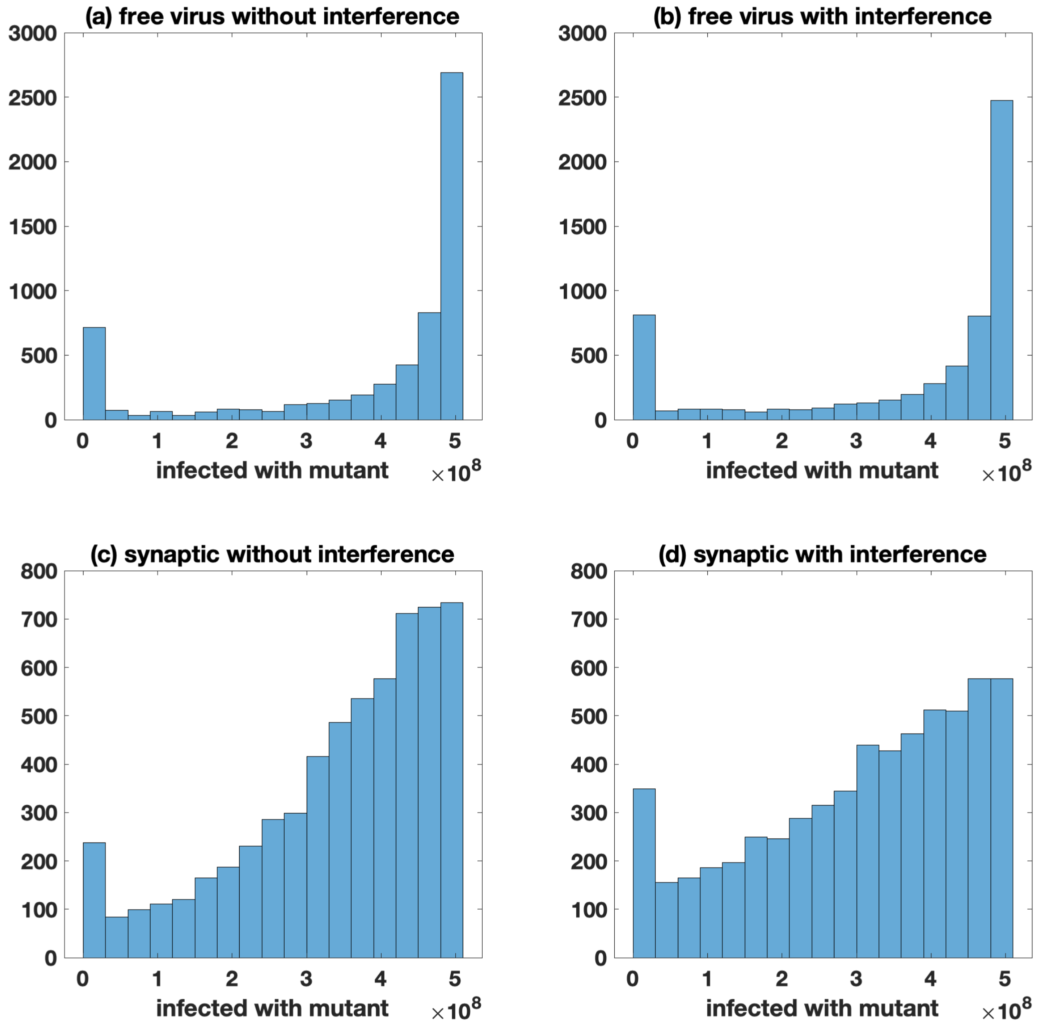


Fig S13: 10% advantageous mutant ($F_{\text{wild-type}} = 0.9, F_{\text{mutant}} = 0.99$), comparing the effect of interference and free virus versus synaptic transmission near peak infection, in the absence of mutations ($\mu = 0$). All simulations start with a single infected cell coinfected with a single copy of both the wild-type and mutant. Panels A and B represent only free virus transmission ($\beta = 3.60 \times 10^{-9},\ \gamma = 0,\ N = 11$), whereas panels C and D represent only synaptic transmission ($\beta = 0,\ \gamma = 3.60 \times 10^{-9},\ N = 25$). The Kolmogorov-Smirnov test between panels A and B and panels C and D gives a $p$-value less than $10^{-6}$. A Only free virus transmission without interference. The average number of cells infected with the mutant is $3.8 \times 10^8$. B Only free virus transmission with interference. The average number of cells infected with the mutant is $3.6 \times 10^8$. C Only synaptic transmission without interference. The average number of cells infected with the mutant is $3.4 \times 10^8$. D Only synaptic transmission with interference. The average number of cells infected with the mutant is $3.1 \times 10^8$. Histograms represent $6 \times 10^3$ hybrid simulations with size threshold $\mathcal{M} = 50$. Simulations in which infections are not established (or in the rare case a simulation does not reach the infected size threshold) are discarded; simulations are stopped when the infected cell population is close to peak infection ($5 \times 10^8$ cells). The other parameters are $\lambda = 1.59 \times 10^7,\ a = 0.45$, and $d = 0.016$.

## References


1. Chun TW, Carruth L, Finzi D, Shen X, DiGiuseppe JA, Taylor H, et al. HIV recombination: what is the impact on antiretroviral therapy? Nature. 1997;387(6629):183–188.

2. Perelson AS, Neumann AU, Markowitz M, Leonard JM, Ho DD. HIV-1 dynamics in vivo: virion clearance rate, infected cell life-span, and viral generation time. Science. 1996;271(5255):1582–1586.

3. Ribeiro RM, Qin L, Chavez LL, Li D, Self SG, Perelson AS. Estimation of the initial viral growth rate and basic reproductive number during acute HIV-1 infection. Journal of virology. 2010;84(12):6096–6102.

4. Nowak MA, Lloyd AL, Vasquez GM, Wiltrout TA, Wahl LM, Bischofberger N, et al. Viral dynamics of primary viremia and antiretroviral therapy in simian immunodeficiency virus infection. Journal of virology. 1997;71(10):7518–7525.

5. Kreger J, Garcia J, Zhang H, Komarova NL, Wodarz D, Levy DN. Quantifying the dynamics of viral recombination during free virus and cell-to-cell transmission in HIV-1 infection. Virus Evolution. 2021;7(1). doi:10.1093/ve/veab026.

6. Pearson JE, Krapivsky P, Perelson AS. Stochastic Theory of Early Viral Infection: Continuous versus Burst Production of Virions. PLOS Computational Biology. 2011;7(2):1–17. doi:10.1371/journal.pcbi.1001058.

7. De Boer RJ, Ribeiro RM, Perelson AS. Current estimates for HIV-1 production imply rapid viral clearance in lymphoid tissues. PLoS computational biology. 2010;6(9):e1000906. doi:10.1371/journal.pcbi.1000906.

8. Rodriguez-Brenes IA, Komarova NL, Wodarz D. The role of telomere shortening in carcinogenesis: A hybrid stochastic-deterministic approach. Journal of Theoretical Biology. 2019;460:144 – 152. doi:https://doi.org/10.1016/j.jtbi.2018.09.003.

9. Whittle P. The outcome of a stochastic epidemic—a note on Bailey's paper. Biometrika. 1955;42(1-2):116–122. doi:10.1093/biomet/42.1-2.116.

10. Allen LJS, van den Driessche P. Relations between deterministic and stochastic thresholds for disease extinction in continuous- and discrete-time infectious disease models. Mathematical Biosciences. 2013;243(1):99 – 108. doi:https://doi.org/10.1016/j.mbs.2013.02.006.

11. Gillespie DT, Petzold LR. Improved leap-size selection for accelerated stochastic simulation. The journal of chemical physics. 2003;119(16):8229–8234.

12. Cao Y, Gillespie DT, Petzold LR. Efficient step size selection for the tau-leaping simulation method. The Journal of chemical physics. 2006;124(4):044109.

13. Gillespie DT. Stochastic Simulation of Chemical Kinetics. Annual Review of Physical Chemistry. 2007;58(1):35–55. doi:10.1146/annurev.physchem.58.032806.104637.

14. Davis MHA. Piecewise-Deterministic Markov Processes: A General Class of Non-Diffusion Stochastic Models. Journal of the Royal Statistical Society: Series B (Methodological). 1984;46(3):353–376. doi:10.1111/j.2517-6161.1984.tb01308.x.

15. Davis MHA. Markov Models and Optimization. Chapman and Hall; 1993.

16. Azaïs R, Bardet JB, Génadot A, Krell N, Zitt PA. Piecewise deterministic Markov process — recent results. ESAIM: Proceedings. 2014;44:276–290. doi:10.1051/proc/201444017.

17. Cloez B, Saporta Bd, Joubaud M. Optimal stopping for measure-valued piecewise deterministic Markov processes. Journal of Applied Probability. 2020;57(2):497–512. doi:10.1017/jpr.2020.18.

18. Abboud C, Senoussi R, Soubeyrand S. Piecewise-deterministic Markov Processes for Spatio-temporal Population Dynamics. In: Statistical Inference for Piecewise-deterministic Markov Processes. John Wiley & Sons, Ltd; 2018. p. 209–255.

19. Lin YT, Buchler NE. Efficient analysis of stochastic gene dynamics in the non-adiabatic regime using piecewise deterministic Markov processes. Journal of The Royal Society Interface. 2018;15(138):20170804. doi:10.1098/rsif.2017.0804.

20. Rudnicki R, Tyran-Kamińska M. Piecewise Deterministic Markov Processes in Biological Models. In: Banasiak J, Bobrowski A, Lachowicz M, editors. Semigroups of Operators - Theory and Applications. Springer Proceedings in Mathematics & Statistics. Cham: Springer International Publishing; 2015. p. 235–255.

21. Zeiser S, Franz U, Wittich O, Liebscher V. Simulation of genetic networks modelled by piecewise deterministic Markov processes. IET systems biology. 2008;2(3):113–135. doi:10.1049/iet-syb:20070045.

22. Lawley SD. Extreme first passage times of piecewise deterministic Markov processes. IOP Science. 2021;34(5):2750–2780. doi:10.1088/1361-6544/abcb07.

23. Faggionato A, Gabrielli D, Ribezzi Crivellari M. Non-equilibrium Thermodynamics of Piecewise Deterministic Markov Processes. Journal of Statistical Physics. 2009;137(2):259. doi:10.1007/s10955-009-9850-x.

24. Lemaire V, Thieullen M, Thomas N. Exact Simulation of the Jump Times of a Class of Piecewise Deterministic Markov Processes. Journal of Scientific Computing. 2018;75(3):1776–1807. doi:10.1007/s10915-017-0607-4.

25. Chraibi H, Dutfoy A, Galtier T, Garnier J. On the optimal importance process for piecewise deterministic Markov process. ESAIM: Probability and Statistics. 2019;23:893–921. doi:10.1051/ps/2019015.

26. Ding S, Qian M, Qian H, Zhang X. Numerical simulations of piecewise deterministic Markov processes with an application to the stochastic Hodgkin-Huxley model. The Journal of Chemical Physics. 2016;145(24):244107. doi:10.1063/1.4971429.

27. Jung A, Maier R, Vartanian JP, Bocharov G, Jung V, Fischer U, et al. Recombination: Multiply infected spleen cells in HIV patients. Nature. 2002;418(6894):144.

28. Josefsson L, King MS, Makitalo B, Brännström J, Shao W, Maldarelli F, et al. Majority of CD4+ T cells from peripheral blood of HIV-1–infected individuals contain only one HIV DNA molecule. Proceedings of the National Academy of Sciences. 2011;108(27):11199–11204.

29. Josefsson L, Palmer S, Faria NR, Lemey P, Casazza J, Ambrozak D, et al. Single cell analysis of lymph node tissue from HIV-1 infected patients reveals that the majority of CD4+ T-cells contain one HIV-1 DNA molecule. PLoS pathogens. 2013;9(6):e1003432.

30. Sigal A, Kim JT, Balazs AB, Dekel E, Mayo A, Milo R, et al. Cell-to-cell spread of HIV permits ongoing replication despite antiretroviral therapy. Nature. 2011;477(7362):95.

31. Komarova NL, Wodarz D. Virus dynamics in the presence of synaptic transmission. Mathematical biosciences. 2013;242(2):161–171.

32. Wodarz D, Levy DN, Komarova NL. Multiple infection of cells changes the dynamics of basic viral evolutionary processes. Evolution letters. 2018;3(1):104–115. doi:10.1002/evl3.95.
