# Supplementary figures and images for: A hybrid stochastic-deterministic approach to explore multiple infection and evolution in HIV
Source: PLoS Comput Biol. 2021 Dec 22;17(12):e1009713. doi: 10.1371/journal.pcbi.1009713 (PMC8730440; doi:10.1371/journal.pcbi.1009713)

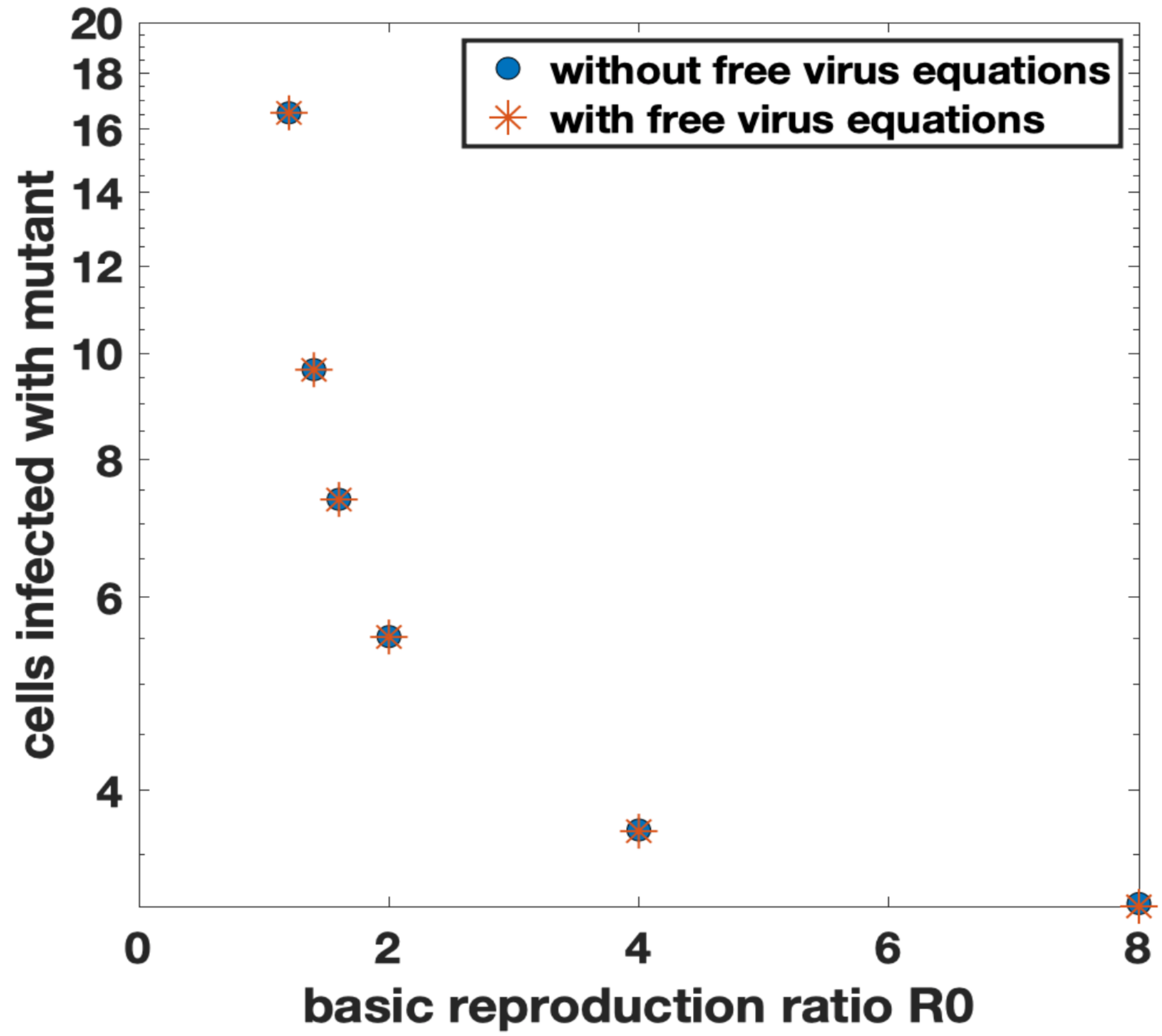

Supplement: S1 Fig — The blue dots represent the deterministic prediction for the number of cells infected with the mutant strain when the total number of infected cells has reached 104 (and are the same as the blue dots in Fig 1A in the main text), in the absence of the explicit free virus equations. The red stars represent the same quantity in the presence of the free virus equations. The parameters are Fwild-type = 0.9, N = 3, μ = 3 × 10^−5^, λ = 1.59 × 10^7^, β = 4 × 10^−9^, γ = 0, k = 2.25 × 10^4^, u = 500, and d = 0.016. The infected cell death rate a is adjusted to achieve the required R0. (PDF) [file pcbi.1009713.s003.pdf]

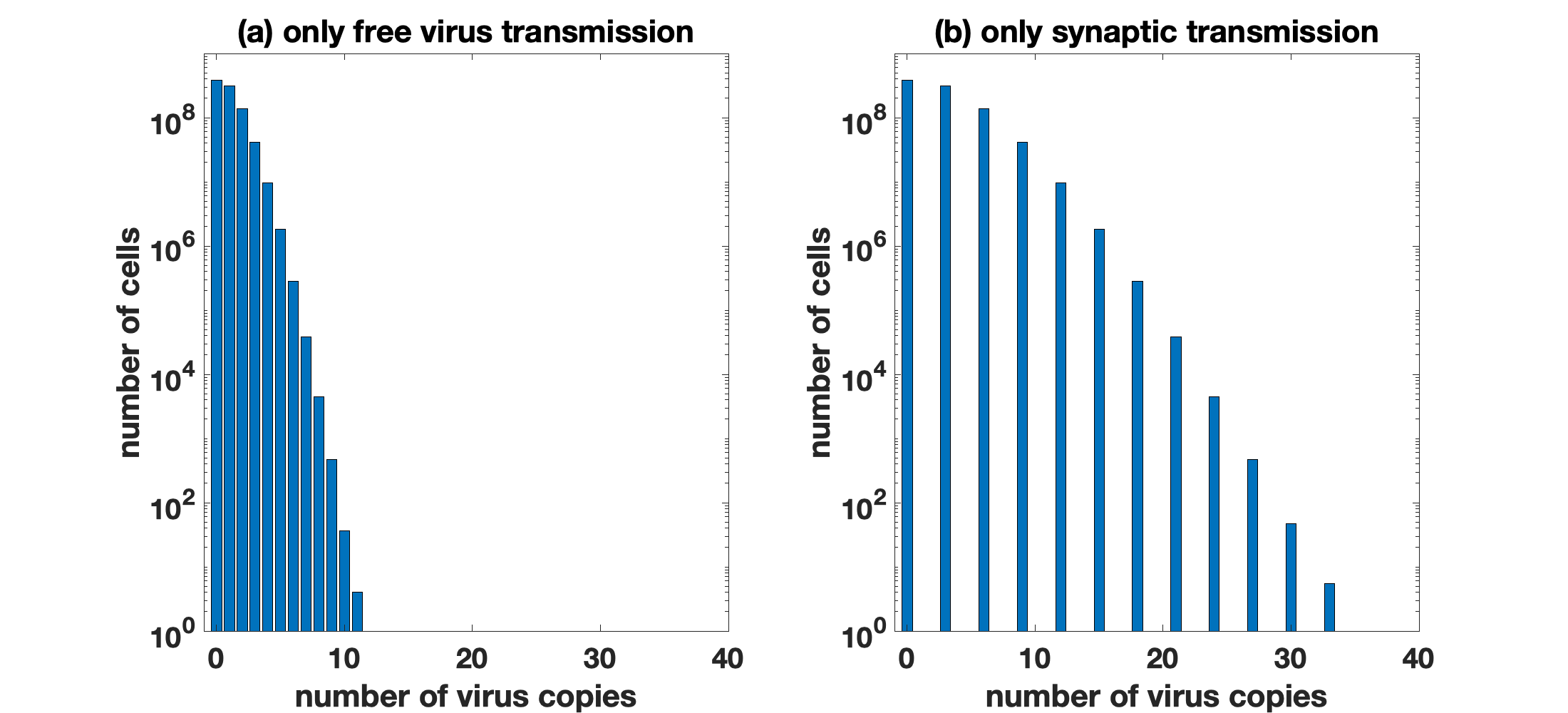

Supplement: S2 Fig — The horizontal axis represents the average number of cells infected with the given number of viral copies, A for only free virus transmission, B for only synaptic transmission. S8 Fig also shows histograms for the average number of cells infected with the given number of viral copies for half free virus transmission and half synaptic transmission. The vertical axis is the average number of cells that are infected with different numbers of viral copies near peak infection. Infected with zero copies corresponds to the uninfected cells. Histograms were averaged over 102 hybrid simulations with size threshold ℳ=50. Simulations are stopped when the infected cell population is near peak infection (5 × 10^8^ cells). Parameters are β + γ = c = 3.6 × 10^−9^, μ = 3 × 10^−5^, λ = 1.59 × 10^7^, a = 0.45, and d = 0.016, and maximum multiplicity of infection N is set to be large enough such that no cells reach this threshold. (PNG) [file pcbi.1009713.s004.png]

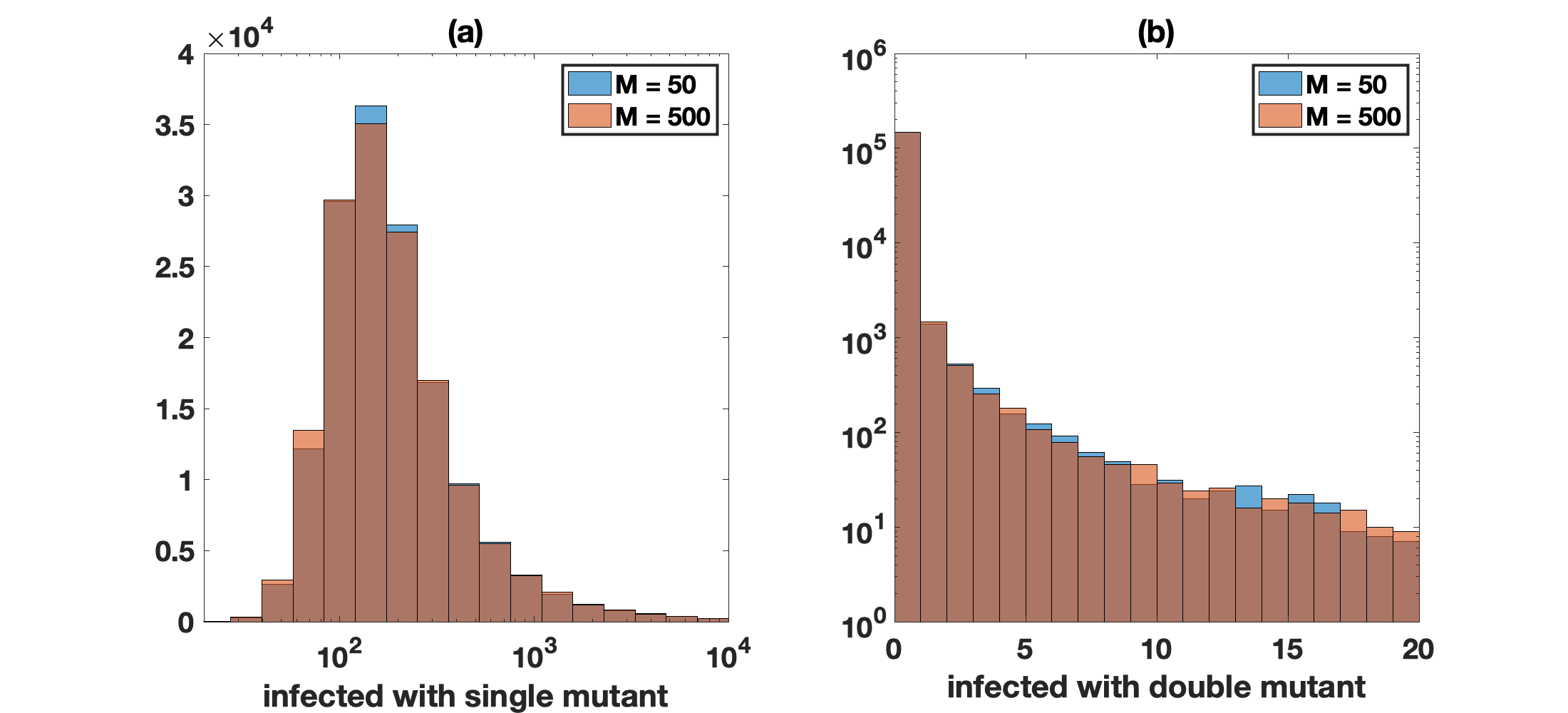

Supplement: S3 Fig — Simulations include only free virus transmission. For each size threshold 1.5 × 10^5^ simulations were performed. A Number of cells infected with one of the single mutant strains. B Number of cells infected with the double mutant strain. The number of cells infected with the double mutant is not statistically different for ℳ=50 and ℳ=500 (p > 0.1 by Kolmogorov-Smirnov test). The parameters are N = 3, μ = 3 × 10^−5^, λ = 1.59 × 10^7^, β = 3.60 × 10^−9^, γ = 0, a = 0.45, d = 0.016, and R0 = 8. (PNG) [file pcbi.1009713.s005.png]

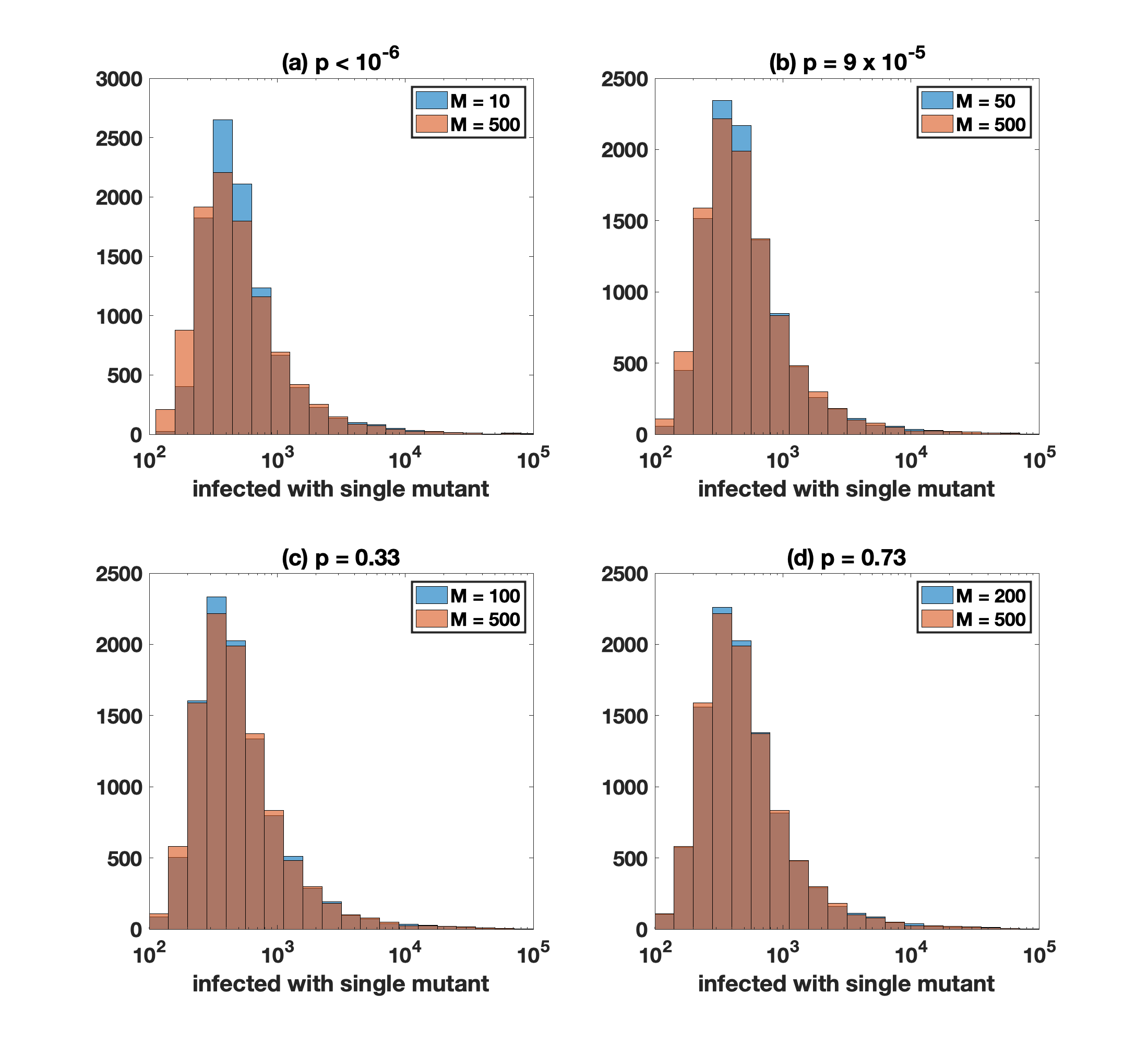

Supplement: S4 Fig — Histograms for each size threshold represent 104 simulations. The p-value from Kolmogorov-Smirnov test is shown for each comparison. A Size threshold ℳ=10 and ℳ=500. B Size threshold ℳ=50 and ℳ=500. C Size threshold ℳ=100 and ℳ=500. D Size threshold ℳ=200 and ℳ=500. Here N = 1 and the other parameters are μ = 3 × 10^−5^, λ = 1.59 × 10^7^, β = 3.60 × 10^−9^, γ = 0, d = 0.016, and R0 = 1.5. (PNG) [file pcbi.1009713.s006.png]

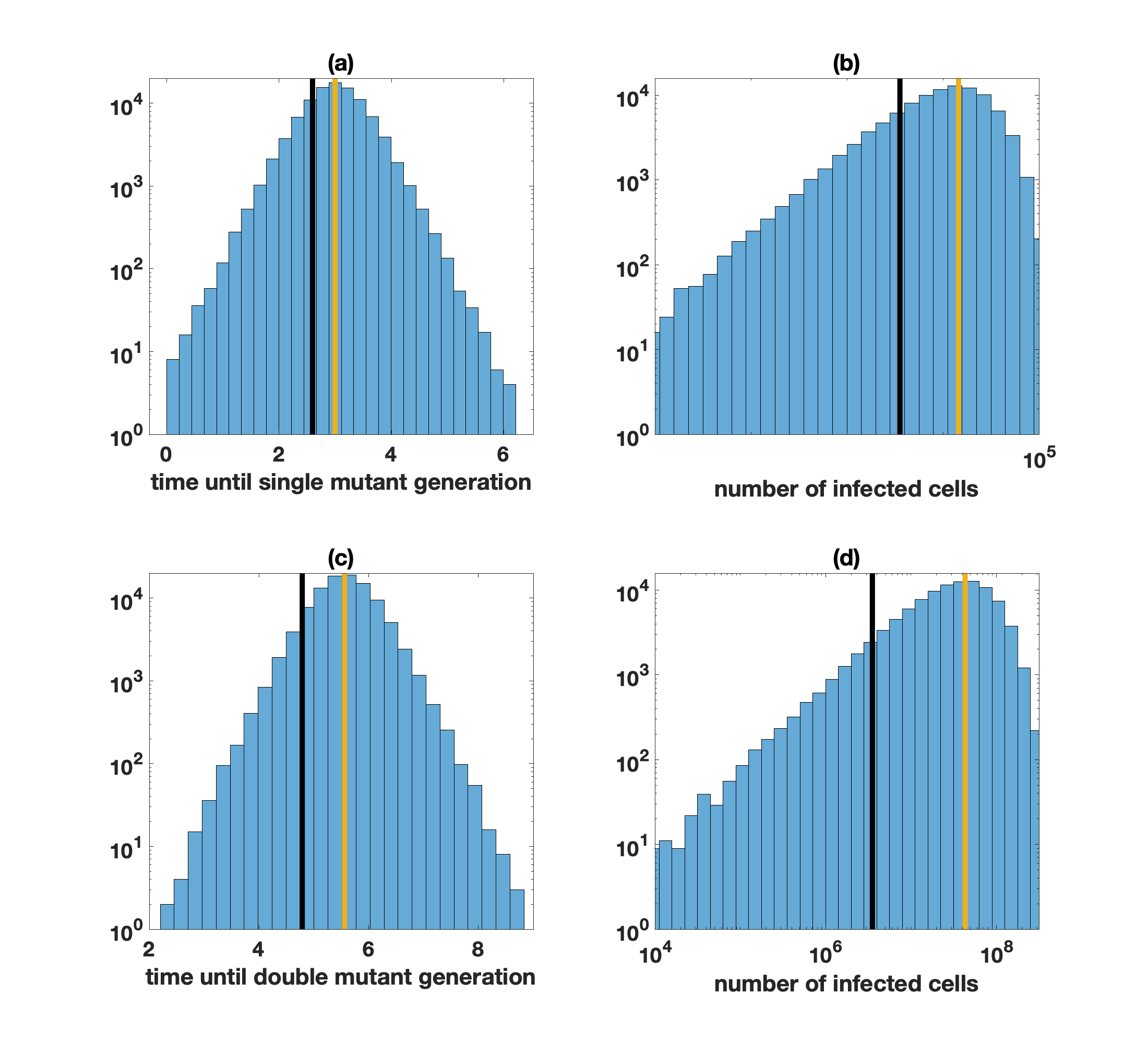

Supplement: S5 Fig — Simulations in which infections are not established are discarded when calculating the averages. The deterministic prediction is denoted with the black vertical line and the hybrid average is denoted with the yellow vertical line. Histograms represent 105 hybrid simulations with size threshold ℳ=50. A Time until either single mutant generation. The deterministic prediction that the first single mutant virus (of both strains) will be generated is around 2.6 days, whereas in the stochastic case it is around 3 days. B Number of infected cells at first single mutant generation. The deterministic prediction is that the number of infected cells is around 3.5 × 10^3^, whereas in the stochastic case it is around 1.4 × 10^4^. C Time until double mutant generation. The deterministic prediction that the first double mutant virus will be generated is around 4.8 days, whereas in the stochastic case it is around 5.6 days. D Number of infected cells at first double mutant generation. The deterministic prediction is that the number of infected cells is around 3.5 × 10^6^, whereas in the stochastic case it is around 4.3 × 10^7^. The parameters are N = 3, μ = 3 × 10^−5^, λ = 1.59 × 10^7^, β = 3.60 × 10^−9^, γ = 0, a = 0.45, d = 0.016, and R0 = 8. (PNG) [file pcbi.1009713.s007.png]

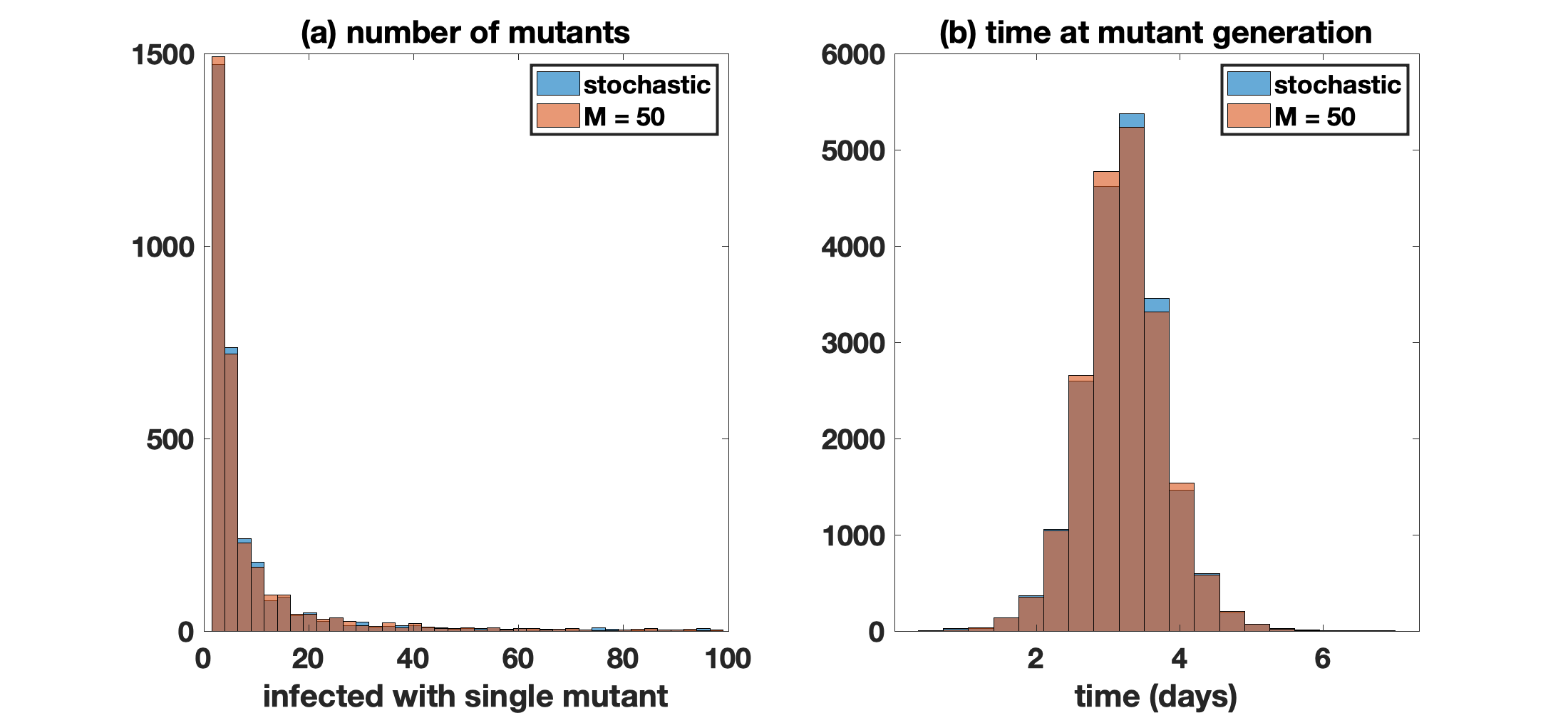

Supplement: S6 Fig — Simulations in which infections are not established are discarded when calculating the averages. Histograms represent 2 × 10^4^ simulations. A The number of cells infected with the mutant at infected population size 104. B The time at first mutant generation. The parameters are Fwild-type = Fmutant = 1, N = 3, μ = 3 × 10^−5^, λ = 1.59 × 10^7^, β = 3.60 × 10^−9^, γ = 0, a = 0.45, d = 0.016, and R0 = 8. (PNG) [file pcbi.1009713.s008.png]

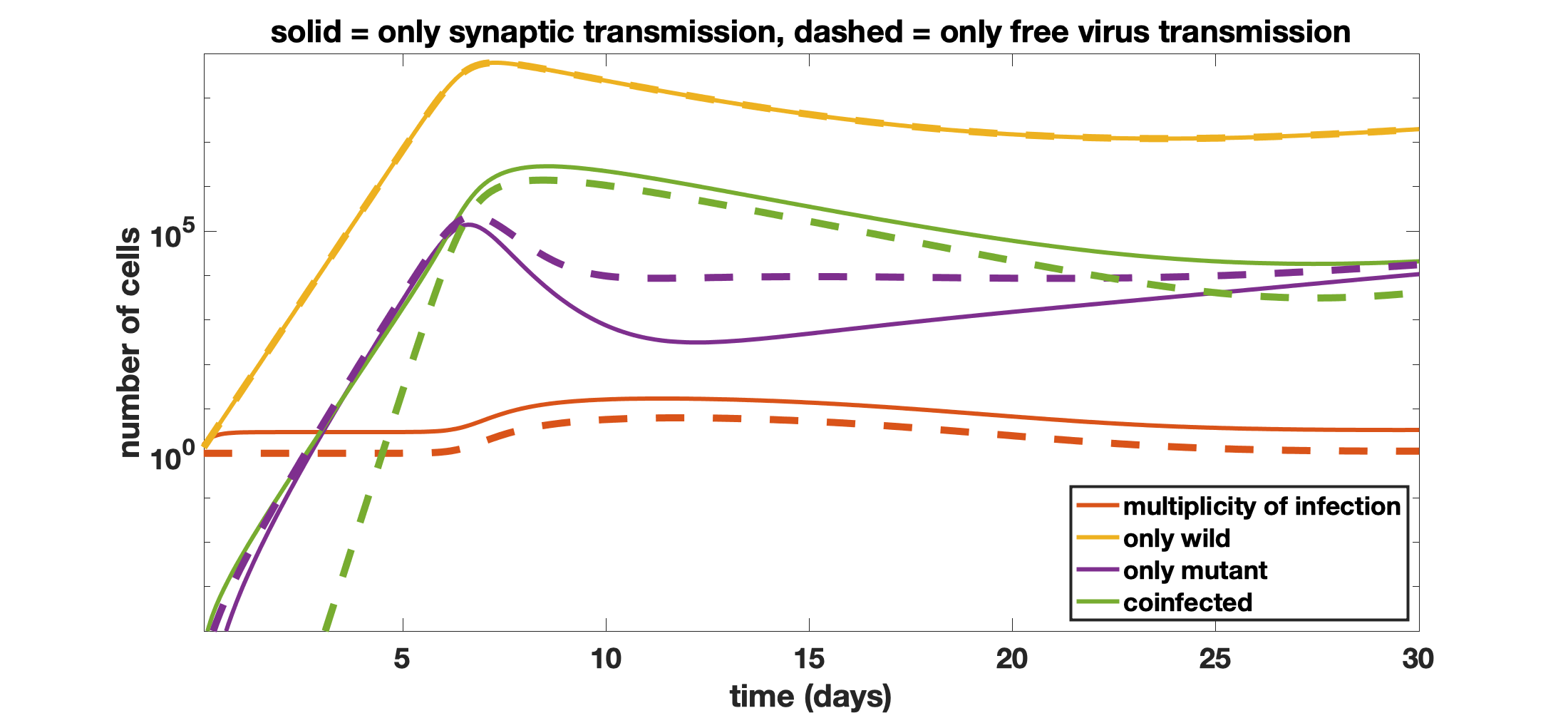

Supplement: S7 Fig — Parameters are N = 25, β + γ = c = 3.6 × 10^−9^, μ = 3 × 10^−5^, λ = 1.59 × 10^7^, a = 0.45, and d = 0.016. The multiplicity of infection is shown with the red lines, the number of cells infected with only the wild-type virus are shown with the yellow lines, the number of cells infected with only the mutant are shown with the purple lines, and the number of cells coinfected with both the wild-type and mutant are shown with the green lines. (PNG) [file pcbi.1009713.s009.png]

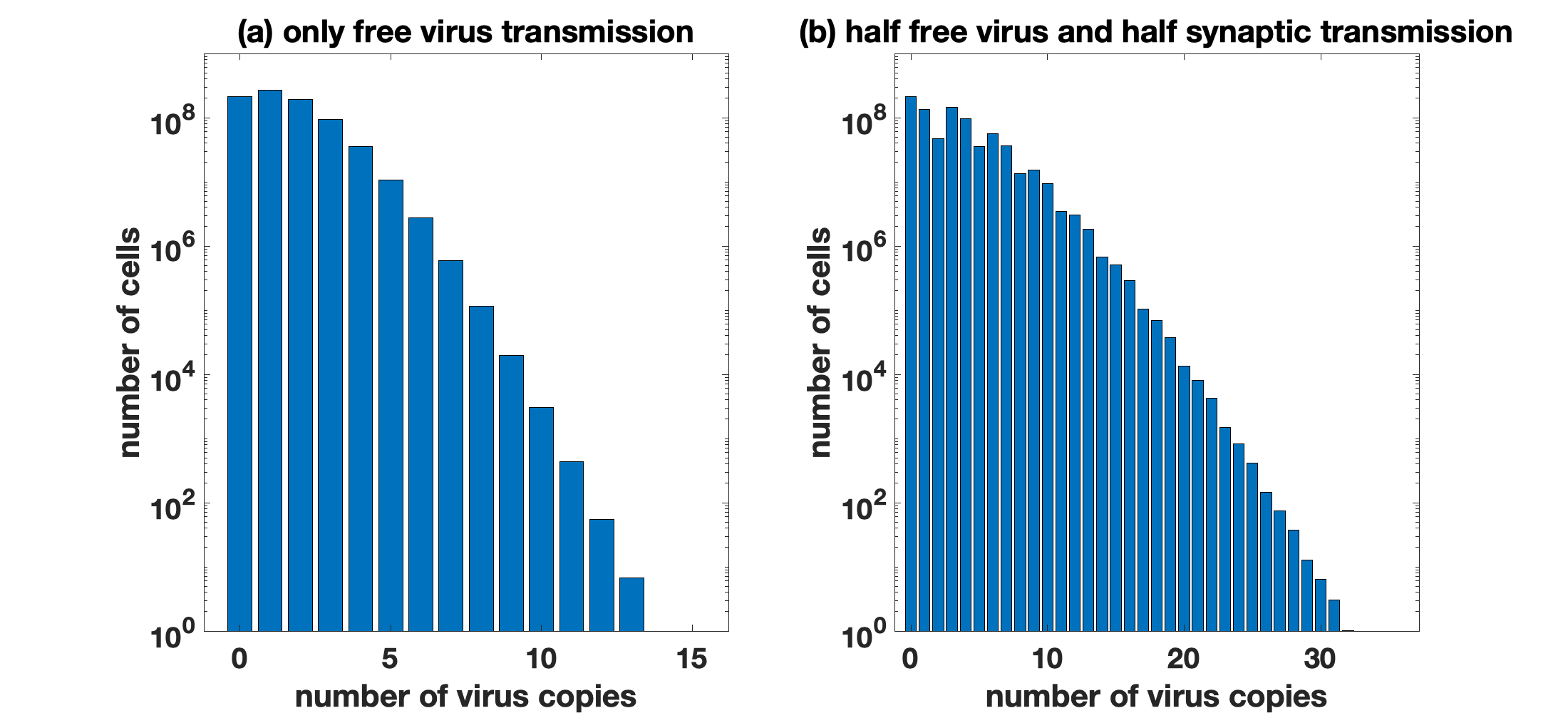

Supplement: S8 Fig — A Histograms for the average number of cells infected with the given number of viral copies for only free virus transmission. B Histograms for the average number of cells infected with the given number of viral copies for half free virus transmission and half synaptic transmission. The horizontal axis is the number of virus copies and the vertical axis is the average number of cells that are infected with that number of viral copies near peak infection. Infected with zero copies corresponds to the uninfected cells. Histograms were averaged over 5 × 10^2^ hybrid simulations with size threshold ℳ=50. Simulations are stopped when the infected cell population is close to peak infection (6 × 10^8^ cells). Parameters are β + γ = c = 3.6 × 10^−9^, μ = 3 × 10^−5^, λ = 1.59 × 10^7^, a = 0.45, and d = 0.016, and maximum multiplicity of infection N is set to be large enough such that no cells reach this threshold. (PNG) [file pcbi.1009713.s010.png]

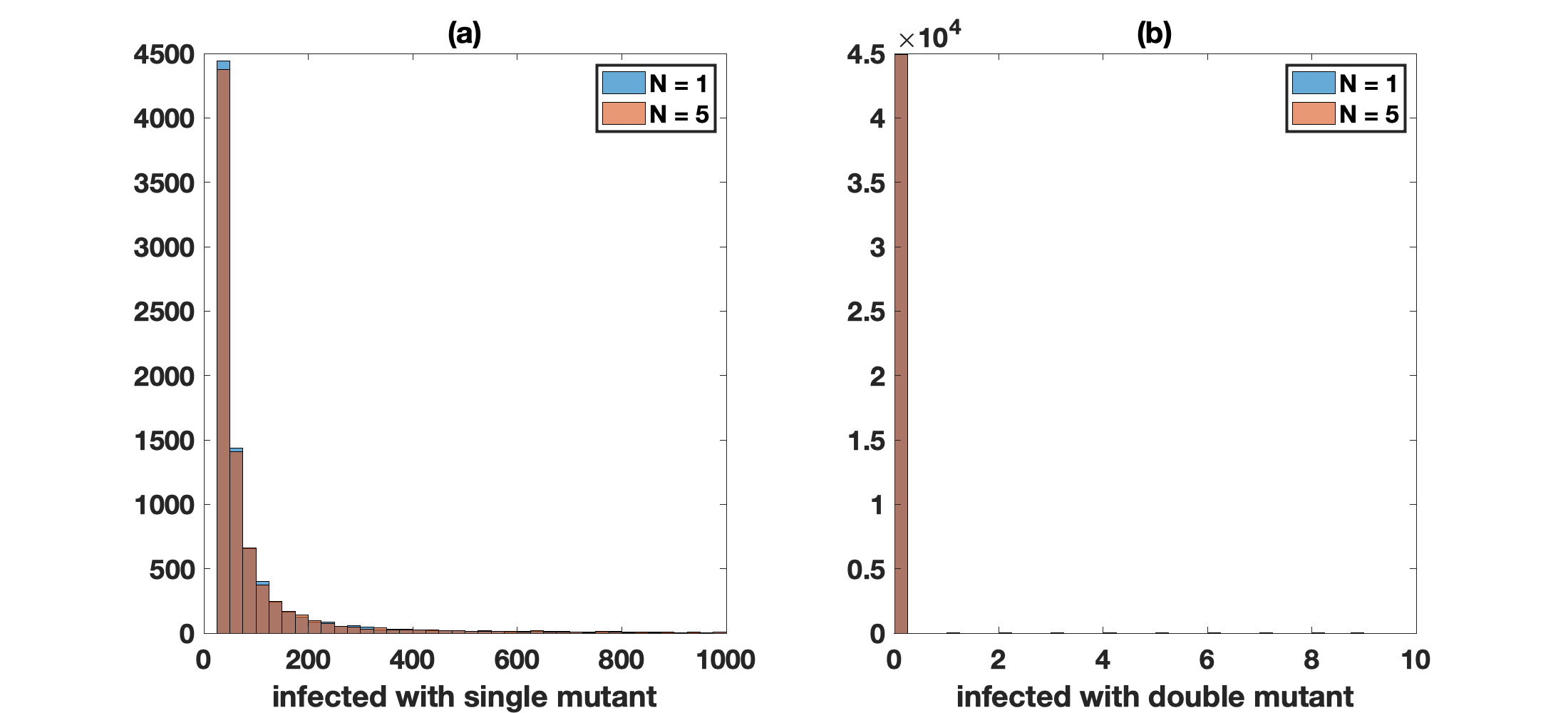

Supplement: S9 Fig — For both panels, the Kolmogorov-Smirnov test between the two distributions suggests that they are not statistically different. A Number of cells infected with one of the single mutant strains. B Number of cells infected with the double mutant strain. Distributions represent 4.5 × 10^4^ hybrid simulations with size threshold ℳ=50. Simulations are stopped at a low viral load (105 cells). The other parameters are as in main text Fig 2 (Fwild-type = 1, Fmutant = 1, μ = 3 × 10^−5^, λ = 1.59 × 10^7^, β = 3.60 × 10^−9^, γ = 0, a = 0.45, and d = 0.016). (PNG) [file pcbi.1009713.s011.png]

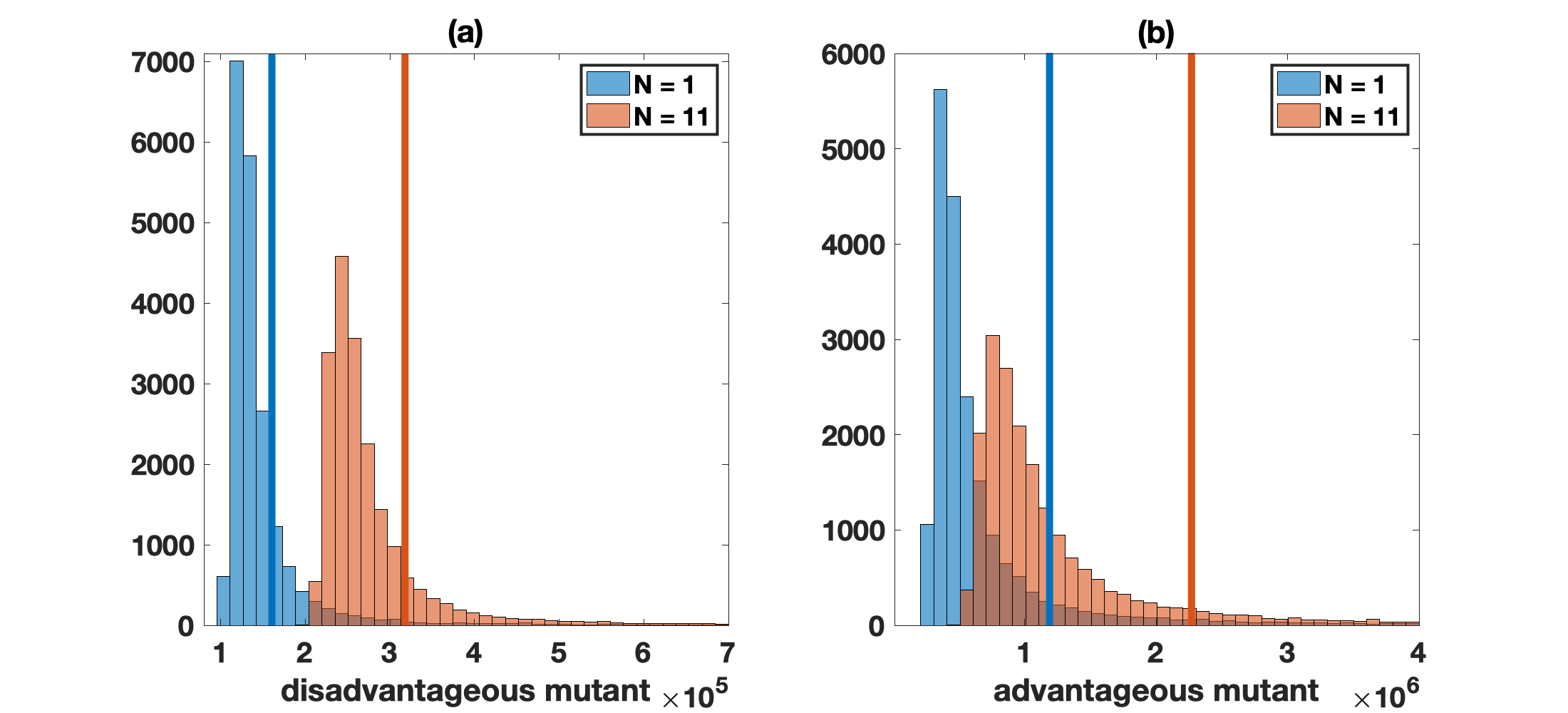

Supplement: S10 Fig — The mean values are shown by black the vertical lines (blue for single infection only and red for multiple infection). For both panels, the Kolmogorov-Smirnov test between the two distributions gives a p-value less than 10−6. A Disadvantageous mutant; here Fmutant = 0.81. The average under single infection only is approximately 1.6 × 10^5^ and for multiple infection is approximately 3.2 × 10^5^. B Advantageous mutant with interference; here Fmutant = 0.99. The average for single infection is approximately 1.2 × 10^6^ and for multiple infection is approximately 2.3 × 10^6^. Histograms represent 2 × 10^4^ hybrid simulations with size threshold ℳ=50. Simulations are stopped when the infected cell population is close to peak infection (6 × 10^8^ cells). The other parameters are Fwild-type = 0.9, μ = 3 × 10^−5^, λ = 1.59 × 10^7^, β = 4 × 10^−9^, γ = 0, a = 0.45, and d = 0.016. (PNG) [file pcbi.1009713.s012.png]

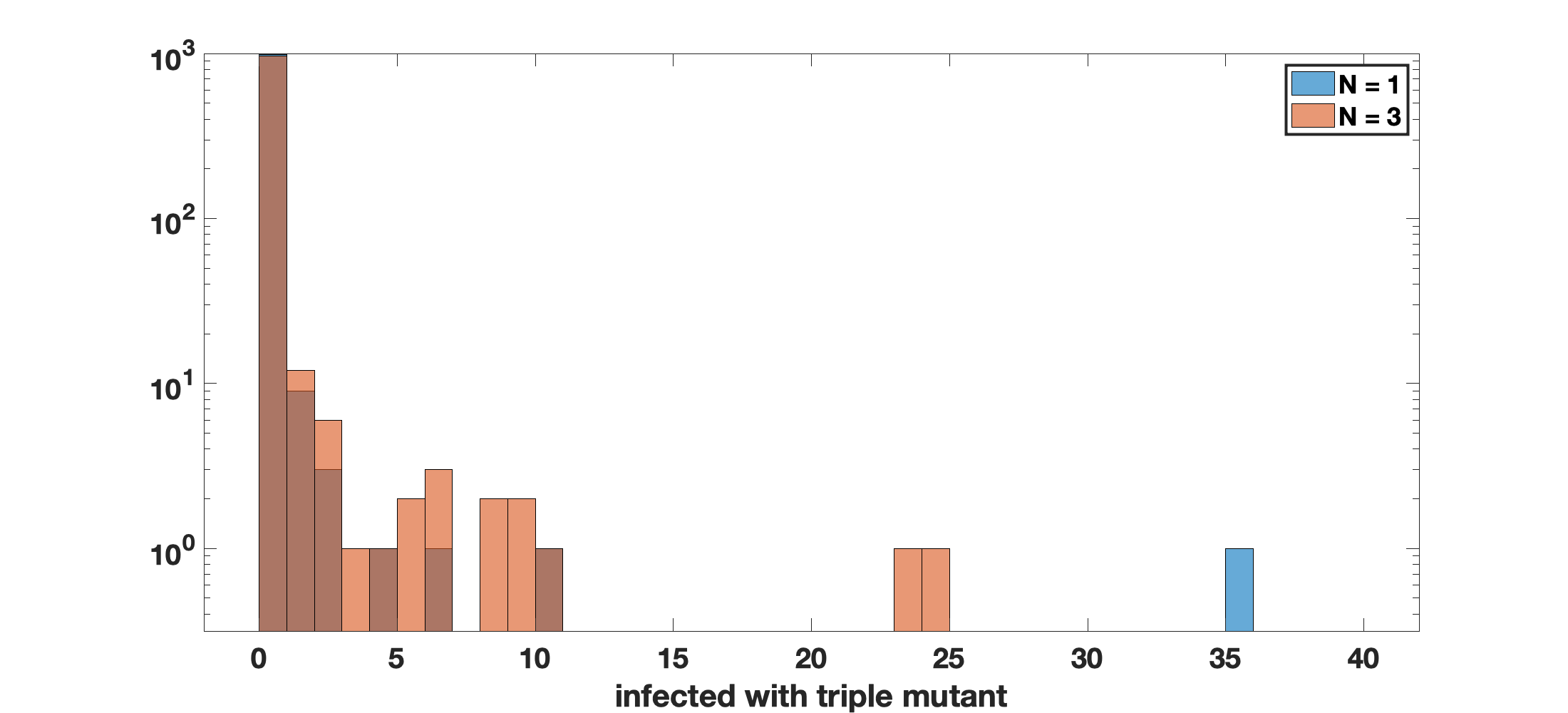

Supplement: S11 Fig — The probability to have at least one cell infected by a triple-mutant is 3.3% under multiple infection, which is about 1.7 times higher than that under single infection (1.9%). This result is significant with p = 2.5 × 10^−3^ by the Z-test, with 2.7 × 10^4^ runs under single infection and 1.9 × 10^3^ runs under multiple infection. Histograms represent 103 hybrid simulations with size threshold ℳ=50. Simulations are stopped when the infected cell population is close to peak infection (6 × 10^8^ cells). All strains are neutral (F = 1) and all other parameters are μ = 3 × 10^−5^, λ = 1.59 × 10^7^, β = 3.60 × 10^−9^, γ = 0, a = 0.45, and d = 0.016. (PNG) [file pcbi.1009713.s013.png]

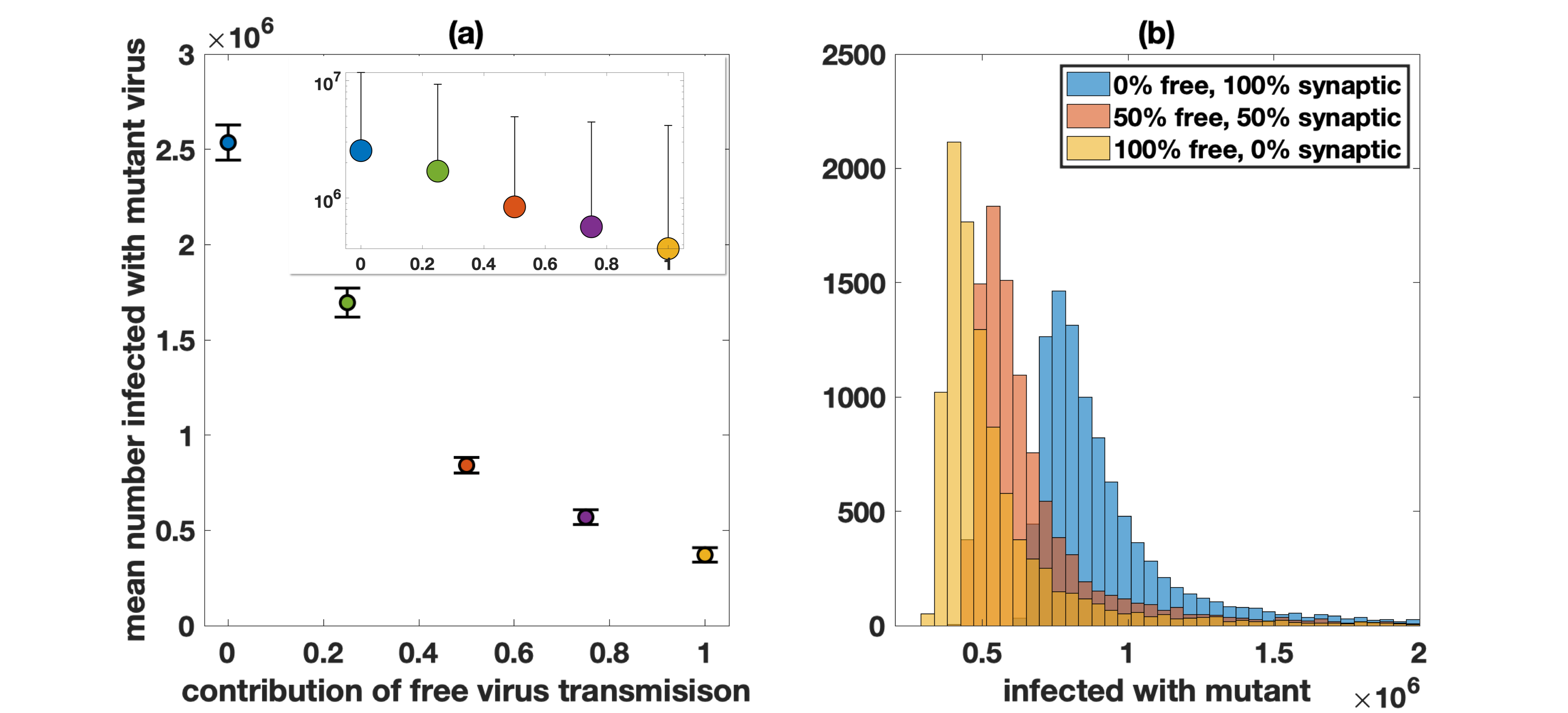

Supplement: S12 Fig — A Number of cells infected with the mutant strain under different transmission strategies. The horizontal axis is the percent contribution of free virus transmission. Standard error bars are shown in the main figure, and standard deviation bars are shown in the inset. B Histograms for the number of cells infected with a single mutant for the different strategies, representing 104 hybrid simulations with size threshold ℳ=50. Simulations are stopped when the infected cell population is close to peak infection (6 × 10^8^ cells) and simulations where no infection is established are thrown out. Here Fwild-type = 1, Fmutant = 1, S = 3, N = 25, β + γ = c = 3.6 × 10^−9^, and the other parameters are μ = 3 × 10^−5^, λ = 1.59 × 10^7^, a = 0.45, and d = 0.016. (PNG) [file pcbi.1009713.s014.png]

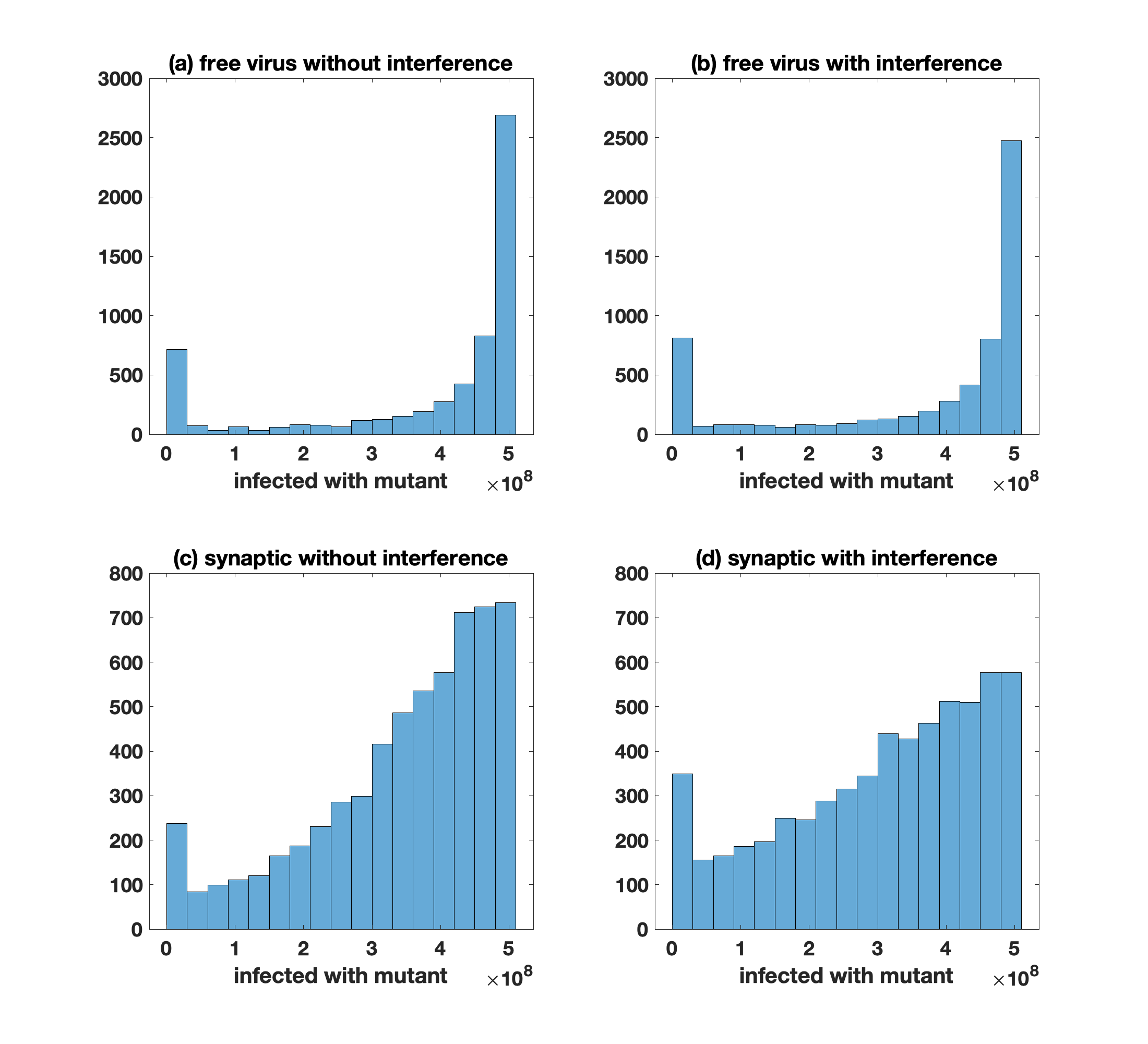

Supplement: S13 Fig — All simulations start with a single infected cell coinfected with a single copy of both the wild-type and mutant. Panels A and B represent only free virus transmission (β = 3.60 × 10^−9^, γ = 0, N = 11), whereas panels C and D represent only synaptic transmission (β = 0, γ = 3.60 × 10^−9^, N = 25). The Kolmogorov-Smirnov test between panels A and B and panels C and D gives a p-value less than 10−6. A Only free virus transmission without interference. The average number of cells infected with the mutant is 3.8 × 10^8^. B Only free virus transmission with interference. The average number of cells infected with the mutant is 3.6 × 10^8^. C Only synaptic transmission without interference. The average number of cells infected with the mutant is 3.4 × 10^8^. D Only synaptic transmission with interference. The average number of cells infected with the mutant is 3.1 × 10^8^. Histograms represent 6 × 10^3^ hybrid simulations with size threshold ℳ=50. Simulations in which infections are not established (or in the rare case a simulation does not reach the infected size threshold) are discarded; simulations are stopped when the infected cell population is close to peak infection (5 × 10^8^ cells). The other parameters are λ = 1.59 × 10^7^, a = 0.45, and d = 0.016. (PNG) [file pcbi.1009713.s015.png]
