# Supplementary material for: A hybrid stochastic-deterministic approach to explore multiple infection and evolution in HIV
Source: PLoS Comput Biol. 2021 Dec 22;17(12):e1009713. doi: 10.1371/journal.pcbi.1009713 (PMC8730440; doi:10.1371/journal.pcbi.1009713)
Supplement: Attachment — Submitted filename: ReviewReport_Kreger.pdf [file pcbi.1009713.s016.pdf]

Review Report: Manuscript "**A hybrid stochastic-deterministic approach to explore multiple infection and evolution in HIV**" by Kreger, Komarova, and Wodarz

In this manuscript, authors presented a hybrid stochastic-deterministic approach to study multiple viral infection and emergence of mutant virus during HIV infection. The authors claim that the presented hybrid approach is computationally efficient to produce results comparable to the fully stochastic dynamics. The advantage of hybrid approach is that it can be used to produce information such as the mutant distribution or the probability of mutant existence at a given infected cell population size, which cannot be obtained from the deterministic ODE-based models. They applied their method to study the role of multiple infection and intracellular interactions among different virus strains (such as complementation and interference) for mutant evolution. The research topic is important and the approach is interesting. However, I have some concerns on the approach, results and their uncertainty, that need to be addressed and/or clarified.

My concerns are:

1. The main concern is the way the viral infection mechanism is introduced in the model, especially the way the infection through cell-free virus is included. Most of the results are concentrated on differentiating cell-free virus infection and synaptic transmission, but mathematically they are modeled in the same way. In the model, cell-free virus (and also synaptic transmission) is modeled via interaction with infected cells. For the proper modeling in the context of this study (i.e., distinguishing two infection mechanisms), free-virus population need to be considered. Quasi-steady state situation might give this approximation, but assuming the quasi-steady state is not appropriate during acute infection and, importantly, in the case of possible synaptic transmission. Without explicitly considering free-virus population, I am not sure that the main result of the paper highlighting different outcomes from these two transmission routes is convincing.
2. It is mentioned that "Mathematically the two processes are similar, except that free virus transmission involves the entry of one virus into the target cells, while multiple viruses (on average S viruses) can enter the target cell simultaneously during synaptic transmission." However, $x_i(t)$ represent the number of cells infected with i copies. Then the free virus transmission term in the model, $\beta x_i(t) x_0$, provides the transmission of i copies. Does not it mean the transmission of i virus (sometime including mutant)? How is it then transmission of one virus in free-virus transmission mechanism? I might have misunderstood. But, in any case, this needs to be clarified. This problem or confusing can be easily resolved adding free-virus population in the model.
3. The manuscript is presented to imply that the deterministic results are not correct because they are not equivalent to the mean of the stochastic results. I don't think authors can claim this based on their current simulations because it could be other way too; the stochastic results are not correct because its mean is not the same as the deterministic results. Only way to figure it out is to compare with the real data. Also, it is not fully clear that this is really true even according to their results. For example, the ODE prediction and stochastic results of the number of cells infected with mutants (Figure 1) are not too different; only a couple of cells difference are observed in most of the cases except for small R_0 in advantageous mutation case. So, I am not sure how we can say that they are indeed different. Similarly, the time until mutant generation and number of infected cells (Figure S3) are almost the same in deterministic and stochastic case (2.6 days vs. 3 days, 4.8 days vs 5.6 days, 3.5×10^3 vs 1.4×10^4, and 3.5×10^6 vs 4.3×10^7). It is not convincing to conclude they are different and deterministic results are not correct. The authors may want to just focus on the hybrid method they developed and produce results,

without claiming that hybrid is better than deterministic. Basically, they both have different purposes for modeling.

4. Figure 3 results: The same value is used for β and γ to compare free-virus case ($\beta = 0$ case) and synaptic case ($\gamma = 0$ case). But, a widely different values of N are used ($N = 11$ and $N = 25$). So, the difference obtained could be because of the different value of N, rather than different mechanism. For fare comparison, the both cases should be simulated for the same value of N. Also, what value of S is used?
5. How to determine δ or pick reasonable value of δ in Equation (12)?
6. The unit of volume is not given. Is it per milliliter? For HIV, the infected cell population size of 5×10^8 seem too high if it is per milliliter. Total CD4 count is 1000 per microliter, which gives 10^6 per milliliter. Moreover, some study (Stafford, JTB, 2000) considers only 1% of this is target of HIV, which makes only 10^4 cells per millimeter are target cells.
7. How is basic reproduction number formula in caption of Figure 1 is derived? In some studies, the basic reproduction number with wild-type and mutant case appears to be $R_0 = \max\{R_0^w, R_0^m\}$, where R_0^w, R_0^m are the basic reproduction numbers corresponding to wild-type and mutants. How is the expression used here compare with previous studies?
