# Supplementary material for: A hybrid stochastic-deterministic approach to explore multiple infection and evolution in HIV
Source: PLoS Comput Biol. 2021 Dec 22;17(12):e1009713. doi: 10.1371/journal.pcbi.1009713 (PMC8730440; doi:10.1371/journal.pcbi.1009713)
Supplement: Attachment — Submitted filename: Kreger_et_al.pdf [file pcbi.1009713.s017.pdf]

Review of Kreger *et. al.*, *A hybrid stochastic-deterministic approach to explore multiple infection and evolution in HIV*

The authors use a hybrid deterministic/stochastic simulation algorithm to model within-host transmission and evolution of HIV. The algorithm switches from a Markov chain to ordinary differential equations as the number infected with viral variants exceeds a threshold determined via the probability of outbreak. In this modelling framework, they show that synaptic transfer of multiple viruses in a single transmission event facilitates greater mutational diversity relative to free transmission and can enable the survival of disadvantageous mutants (which might carry resistance mutations), that triple mutants (which could potentially escape combination therapy) are unlikely, and that interference is relatively unimportant unless fitness benefits are large. There are interesting results, but I believe that the manuscript could benefit from substantial revision.

While I understand the desire to present the simplest possible version of the model in the main text, much of the subsequent discussion centres on mutant strains, their fitnesses *etc.* which remain undefined except in the SI, so the net result is less, not more, clear (*e.g.* the caption to Figure 1 only makes sense if one has read the SI). There should be some reasonable compromise between the level of detail given in the SI and the almost none given in the main text. I also found that the exposition could be improved in places (*e.g.* "a disadvantageous mutant is assumed to have an increased probability that successful infection fails" is a tortuous way of saying "a disadvantageous mutant has a decreased probability of infection").

With the emphasis put on the algorithm (*e.g.* the title) rather than the biological results, it seems to me that the authors should explain/emphasize better how it is novel as compared to the authors' previous work (cited here as [34]) and other multi-scale approaches that have been used in systems biology. Is it the application to an epidemiological model rather than a cancer model? Further, since the virtue of using thresholds and ODEs is speed (at the price of approximation), it would seem more fair to compare performance to widely-used refinements such as *e.g.* adaptive tau-leaping ([3] is a good summary), rather than Gillespie's original algorithm from over forty years ago.

The importance of simultaneous synaptic transmission for most evolutionary effects observed in the modelling call into question the well-mixed assumptions made here; it seems like more spatially

explicit model (which in turn may not be amenable to ODE approximation due to small neighbourhood sizes) with synaptic transmission to nearby cells might not arrive at similar conclusions. A spatial model is mentioned in the discussion, but it would be useful to really understand how robust the conclusions are to mass-action assumptions.

Finally, I would also like to bring to the authors' attention some previous (theoretical) work on multi-scale analysis of stochastic models, in particular [2], which illustrates how one can identify scales and approximations for a viral kinetics model. One of the authors (Tom Kurtz) has decades of work on the subject of stochastic chemical kinetics and approximations, including a recent book [1] which gives a good overview of the subject and an ample bibliography.

Other comments & questions:

- It would benefit potential readers not familiar with HIV dynamics to briefly define/distinguish free *vs.* synaptic transmission when they appear at the top of page 2 (incidentally, adding line numbers via the package *lineno* would simplify life for your reviewers).
- Please define S in the paragraph preceding equations (1) – (5). Any reader familiar with epidemiological models recognizes λ, β and γ, and can thus understand these without need to refer to the table. That is not true of S, which is an important component of the model structure.
- In equations (2) and (4), writing $Z(-x_i)$ and $Z(x_{i-S})$ give the reader that the function Z is being evaluated at $-x_i$ and x_{i-S} respectively (and by extension, Z is being evaluated at the quantities in brackets elsewhere). It would be clearer to write $-\gamma Z x_i$ and $+\gamma Z x_{i-S}$.
- The extinction probability $\frac{1}{R_0}$ for a novel strain (used here to determine the threshold $\mathcal{M}$) is only valid at the disease-free equilibrium (all cells susceptible); how does this affect the choice of thresholds for mutant strains?
- "This is because at time t_N, while the average trajectory reaches size N, some stochastic trajectories at that time will be lower and some higher than N." The trajectories are multi-dimensional; it would be better here to speak of the number infected.
- Results are presented for the exponential growth phase. Do the authors account for the possibility that the number infected will fall below the threshold as a part of the boom-bust dynamics inherent in the model?
- Figure 2 displays histograms, not distributions (the latter are normalized).

Supplementary Information

- What mutation is assumed? I wonder how likely it is that multiple sites segregate simultaneously as opposed to sequentially?
- For the event notation, please indicate that the triples indicate outcomes for the three viruses. Writing the sets of events in brackets gives the impression of quadruples; $(***, **X, *XX, XXX)$, $(**0, *X0, XX0, *00, X00, 000)$ would be perhaps more clearly indicated by $\{***, **X, *XX, XXX\}$ and $\{**0, *X0, XX0, *00, X00, 000\}$.
- Equation (2): I fail to see what is gained by representing a polynomial as a hypergeometric function. Is this done for the purposes of coding?
- I don't know what the phrase "Everything in Equations (14)-(16) is stochastic." is intended to say when applied to an ordinary differential equation. I'm assuming the ODE is meant as a shorthand for the jump rates of a continuous-time Markov chain, but that seems to more confuse than clarify.
- Figure S1 (b): can you explain the tai behaviour (what looks like a second peak at around 10) in the hybrid simulations?

References

[1] D. F. Anderson and T. G. Kurtz. *Stochastic analysis of biochemical systems.* Springer, 2015.

[2] K. Ball, T. G. Kurtz, L. Popovic, and G. Rempala. Asymptotic analysis of multiscale approximations to reaction networks. *Ann. Appl. Probab.*, 16(4):1925–1961, 2006.

[3] D. T. Gillespie. Stochastic simulation of chemical kinetics. *Ann. Rev. Phys. Chem.*, 58(1):35–55, 2007.
