# Supplementary material for: A hybrid stochastic-deterministic approach to explore multiple infection and evolution in HIV
Source: PLoS Comput Biol. 2021 Dec 22;17(12):e1009713. doi: 10.1371/journal.pcbi.1009713 (PMC8730440; doi:10.1371/journal.pcbi.1009713)
Supplement: Attachment — Submitted filename: Review response.pdf [file pcbi.1009713.s018.pdf]

A hybrid stochastic-deterministic approach to explore multiple infection and evolution in HIV

Author responses and changes

We would like to thank the editors and the reviewers for carefully reading our manuscript and coming up with a number of insightful suggestions. We have taken all of the reviewers' points into account and have changed the manuscript accordingly. Among the changes, we have now restructured the manuscript to (1) better describe the novelty and advantages of our approach, specifically for the viral dynamics simulations, and (2) describe the mathematical model in detail in the main text. Also, we have performed additional simulations, e.g. to demonstrate that including free virus dynamics explicitly does not make a significant difference for the system in question, and to show the distribution of multiplicity of infection under different assumptions on the transmission pathway.

Below are our detailed responses and changes. The reviewers' comments are in blue and our responses are in black. Changes in the main text and supplement are shown in blue font.

Reviewer: 1

This paper describes a hybrid stochastic/deterministic approach applied to HIV. The underlying model studied takes into account synaptic cell-to-cell transmission (for multiple strain infections), and neutral, and advantageous/disadvantageous mutations. ODE models are widely adopted to study viral dynamics, despite their downsides when it comes to rare mutational events. The approach described herein therefore has the potential for impact, given that it addresses such events during acute infection, as described in this paper.

1) This paper would benefit significantly from reorganization. If the emphasis is on the computational method, then parts of the SI should be moved to the main paper. I believe this would help the reader better grasp the approach. I would suggest at a minimum that the first section of the SI (full description of the mathematical model) be moved into the main paper, to give more context to the later sections. As written, the main takeaways and the focus of the paper aren't clearly distinguishable.

We thank the referee for this suggestion. In the new version of the manuscript, we present the details of the mathematical modeling in the main text, thus making the mathematical part of the manuscript more coherent.

2) The authors should touch upon why cells non-dimensionalized in the model. Would this be necessary for all implementations of the hybrid approach? Later in figure legends, cells are expressed in cells. If cell units are not not non-dimensionalized, than the units in Table 1 aren't correct.

Variables describing cell populations are measured in terms of cell numbers (rather than concentrations). This choice was made because it allows for easy interpretation of stochastic data (which track every cell in small populations). A note has been inserted in Table 1 of the main text to clarify the units.

3) A more detailed discussion on other hybrid approaches should be undertaken in the introduction. These include, but aren't limited to, tau-leaping and fast-slow systems.

We have included a more detailed discussion on other hybrid approaches including tau-leaping and multi-scale methods in Section 2.3 of the new version of the manuscript, see also the new Section 2.4 of the Supplementary Information for a more detailed comparison with the tau-leaping method. We have also provided more detail on the methodology and selection of our hybrid algorithm.

We first describe why we turn to a hybrid algorithm, which is because as the population size increases the average time step in the Gillespie method decreases, which results in a very computationally inefficient method. In particular, we turn to the method developed in our previous work (Rodriguez-Brenes et al. 2019 Journal of Theoretical Biology, cited previously as [34]), which was specifically developed to handle large population dynamic models where stochastic interactions that effect both very small and very large populations exist (such as random and rare mutation) in an intuitive and computationally efficient way.

We then describe how our hybrid method is based on work in the field of chemical kinetics, which arose from the necessity to simulate systems with a large number of reactants (and so the basic Gillespie method is computationally inefficient). Especially in the field of physical chemistry, many innovative computational algorithms have been developed to simulate stochastic systems, including the next reaction method and tau-leaping/adaptive tau-leaping methods. Here, large computational advantages over the Gillespie method are feasible because of the ability to make large time steps through measuring how many times each stochastic reaction "fires" in the relevant time interval. However, the existence of both small and large populations of importance generally implies that methods such as tau-leaping will be inefficient and furthermore, measuring how many times a reaction "fires" in a given time interval is somewhat counterintuitive when subpopulations vary over several orders of magnitude (Gillespie 2007 Ann. Rev. Phys. Chem, Cao and Petzold 2008 Comput. Methods. Appl. Mech. Eng). To this end, there has also been a focus on the development (as well as mathematical analysis) of novel hybrid stochastic-deterministic approaches, including many different mutli-scale methods (such as fast-slow methods) that are designed to simulate systems that contain different time, size, and spatial scales. While these approaches are often used in physical chemistry, they are less common in the fields of population dynamics and evolution, potentially because they can rely on theoretical concepts such as Langevin's equation which are less common in these fields. Therefore, in this paper, we choose to adapt our previously developed hybrid framework, as our system contains a large

overall population size and number of reactions, random and rare mutation events, and the simultaneous existence of both small and large populations of importance.

More details and references are given in Section 2.3 of the revised main text.

4) Why is free virus not explicitly included in the model (Eqs. 1-5)? As written, the rate of free virus infection is proportional to beta and interactions between uninfected and all infected cells, why not include an equation for virions?

We do not explicitly include free virus equations in the model as we assume that the free virus population is at quasi-steady state and therefore that the rate of free virus transmission is proportional to the interactions between infected and target cells. This is a reasonable assumption as the time-scale of virus spread is long compared to the very quick time-scale of the free virus particles (denoted by v). In the new version of the manuscript we have verified this explicitly for our model by comparing simulations with and without the explicit free virus equations, which is described below (see Section 1.2 of the new version of the Supplement).

This figure (also see the new Figure S1) compares the ordinary differential equation (ODE) prediction in Figure 1(a) in the main text to the corresponding system including explicit free virus equations (numerical values are also included in the table after the figure). An explanation of how to incorporate the free virus equations is provided in Section 1.2 of SI. The blue dots represent the deterministic prediction for the number of cells infected with the mutant strain when the total number of infected cells has reached 10^4 (and are the same as the blue dots in Figure 1(a) in the main text), in the absence of the explicit free virus equations. The red stars represent the same quantity in the presence of the free virus equations.

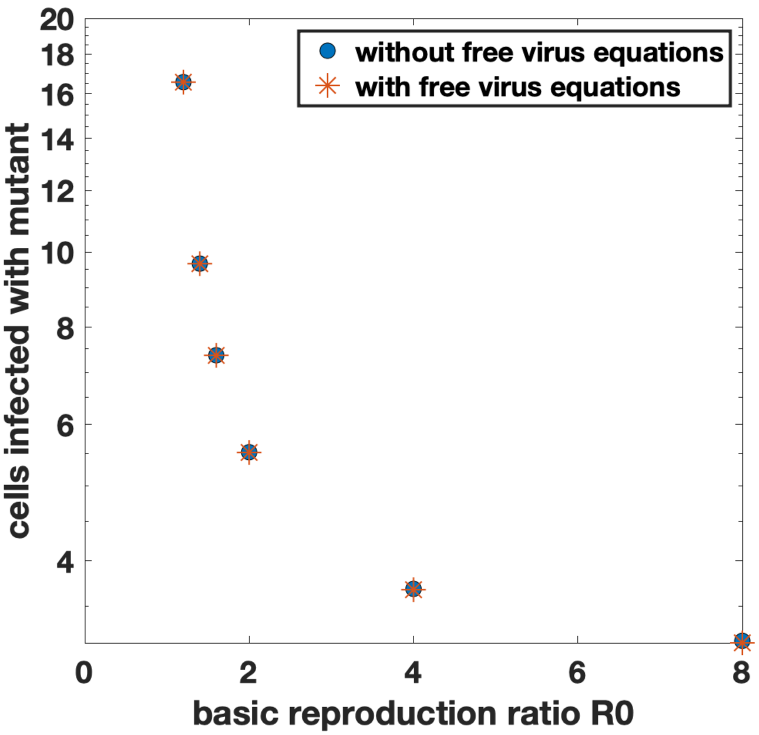

R_0	without free virus equations	with free virus equations	% difference
1.2	16.5517	16.5504	0.00792666
1.4	9.66162	9.65915	0.025534
1.6	7.36284	7.3597	0.0425923
2	5.52304	5.51879	0.0771133
4	3.6821	3.67332	0.238234
8	3.15388	3.13771	0.51267

Table: Numerical values for Figure S1 in SI, comparing deterministic predictions for the number of cells infected with the mutant with/without free virus equations.

We find that if R_0 is closer to 1, then the ODE predictions with/without explicit free virus equations are almost exactly the same (because the time-scale of virus spread is so long compared with the time-scale of the free virus, v). For HIV we have $R_0 = 8$, and the ODE predictions with explicit free virus equations result in a slightly different mean (about a 0.5% difference), because the virus doesn't quite catch up with y on the time-scale of the spread. This demonstrates that the assumption that the free virus population is at quasi-steady state is reasonable and therefore we assume that the rate of free virus transmission is proportional to the interactions between infected and target cells. This is now described in detail in the new Section 1.2 (see also new Figure S1 and Table 1) of the Supplement.

5) Figure 1 and similar figures: the dots are quite large and the inner panel is doubling up information. Since multiple runs of the stochastic algorithm are needed, it would be better to always include mean + SD (or some other metric of dispersion). I would suggest remaking these large dots with another smaller marker and tails.

The dot size in Figure 1 in the main text has been reduced. The main figure shows the deterministic predictions (blue dots) with the stochastic averages (yellow dots) which are presented with standard error bars, which can be too small to be seen. The inner panels show the mean with the standard deviation. This has been additionally clarified in the figure caption. Similar adjustments have also been made to Figure S12 in the Supplementary Information.

6) Figure 2: What do the royal blue and bright red single lines represent?

In Figure 2 in the main text, the mean value of cells infected with the single mutant (panel a) and double mutant (panel b) are denoted by the single vertical lines, where blue denotes single infection only and red denotes multiple infection. This has been additionally clarified in the figure caption as well as for Figure S10 in the Supplementary Information.

Reviewer: 2

1. The main concern is the way the viral infection mechanism is introduced in the model, especially the way the infection through cell-free virus is included. Most of the results are concentrated on differentiating cell-free virus infection and synaptic transmission, but mathematically they are modeled in the same way. In the model, cell-free virus (and also synaptic transmission) is modeled via interaction with infected cells. For the proper modeling in the context of this study (i.e., distinguishing two infection mechanisms), free virus population need to be considered. Quasi-steady state situation might give this approximation, but assuming the quasi-steady state is not appropriate during acute infection and, importantly, in the case of possible synaptic transmission. Without explicitly considering free-virus population, I am not sure that the main result of the paper highlighting different outcomes from these two transmission routes is convincing.

We do not explicitly include free virus equations in the model as we assume that the free virus population is at quasi-steady state and therefore that the rate of free virus transmission is proportional to the interactions between infected and target cells. This is a reasonable assumption as the time-scale of virus spread is long compared to the very quick time-scale of the free virus particles (denoted by v). In the new version of the manuscript we have verified this explicitly for our model by comparing simulations with and without the explicit free virus equations, which is described below (see Section 1.2 of the new version of the Supplement).

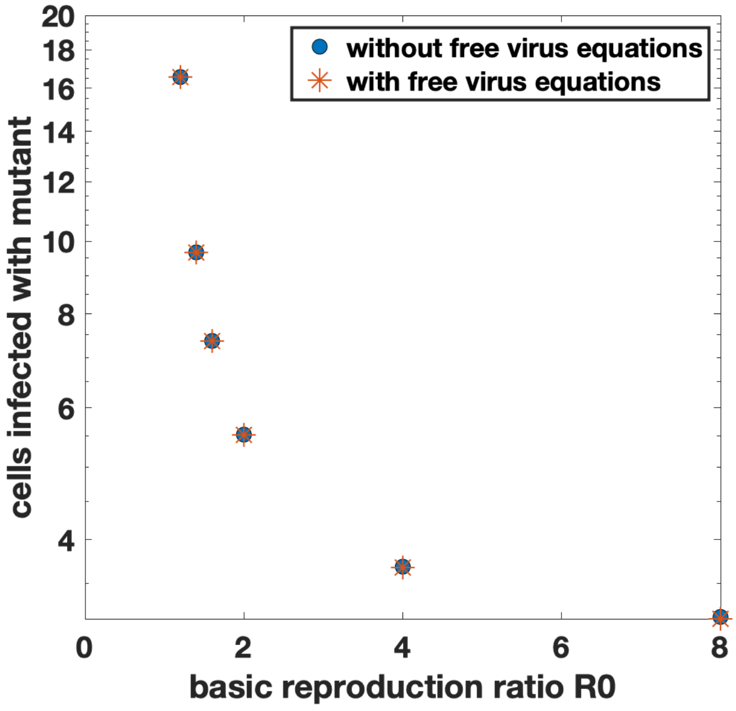

This figure (also see the new Figure S1) compares the ordinary differential equation (ODE) prediction in Figure 1(a) in the main text to the corresponding system including explicit free virus equations (numerical values are also included in the table after the figure). An explanation of how to incorporate the free virus equations is provided in Section 1.2 of SI. The blue dots represent the deterministic prediction for the number of cells infected with the mutant strain when the total number of infected cells has reached 10^4 (and are the same as the blue dots in Figure 1(a) in the main

text), in the absence of the explicit free virus equations. The red stars represent the same quantity in the presence of the free virus equations.

R_0	without free virus equations	with free virus equations	% difference
1.2	16.5517	16.5504	0.00792666
1.4	9.66162	9.65915	0.025534
1.6	7.36284	7.3597	0.0425923
2	5.52304	5.51879	0.0771133
4	3.6821	3.67332	0.238234
8	3.15388	3.13771	0.51267

Table: Numerical values for Figure S1 in SI, comparing deterministic predictions for the number of cells infected with the mutant with/without free virus equations.

We find that if R_0 is closer to 1, then the ODE predictions with/without explicit free virus equations are almost exactly the same (because the time-scale of virus spread is so long compared with the time-scale of the free virus, v). For HIV we have $R_0 = 8$, and the ODE predictions with explicit free virus equations result in a slightly different mean (about a 0.5% difference), because the virus doesn't quite catch up with y on the time-scale of the spread. This demonstrates that the assumption that the free virus population is at quasi-steady state is reasonable and therefore we assume that the rate of free virus transmission is proportional to the interactions between infected and target cells. This is now described in detail in the new Section 1.2 (see also new Figure S1 and Table 1) of the Supplement.

2. It is mentioned that "Mathematically the two processes are similar, except that free virus transmission involves the entry of one virus into the target cells, while multiple viruses (on average S viruses) can enter the target cell simultaneously during synaptic transmission." However, $x_i(t)$ represent the number of cells infected with i copies. Then the free virus transmission term in the model, $\beta x_i(t)\, x_0$ provides the transmission of i copies. Does not it mean the transmission of i virus (sometime including mutant)? How is it then transmission of one virus in free-virus transmission mechanism? I might have misunderstood. But, in any case, this needs to be clarified. This problem or confusing can be easily resolved adding free-virus population in the model.

It is correct that $x_i(t)$ denotes the number of cells infected with i copies of virus. However, the term $\beta x_i(t)\, x_0$ does not indicate transmission of i copies of virus. Please consider Equations (1) and (2) of the main text, take $i = 1$, and note the terms that multiply β. The negative β-term in Equation (1) describes a reduction of uninfected cells, and these cells become singly-infected cells (x_1) through an identical positive term in

Equation (2). This term represents the rate at which infected cells Z (which comprise all subpopulations infected with i =1,2,… copies of virus) infect uninfected cells (x_0), by free virus transmission; again, it is assumed that in quasi-steady state the number of free viruses is proportional to the total population size of infected cells, Z. Similarly, singly infected cells x_1 become superinfected and enter the population $x_{2,}$ and so on, giving rise to the cascade of Equations (1-5).

Here we assume that upon free-virus transmission, a single virus infects the target cell, resulting in a cell of type x_i becoming a cell of type x_{i+1}. Synaptic transmission is mathematically similar, except up to S virus copies are assumed to be transmitted simultaneously, resulting in a cell of type x_i becoming a cell of type x_{i+S}.

This has been clarified in Section 2.1 in the main text.

3. The manuscript is presented to imply that the deterministic results are not correct because they are not equivalent to the mean of the stochastic results. I don't think authors can claim this based on their current simulations because it could be other way too; the stochastic results are not correct because its mean is not the same as the deterministic results. Only way to figure it out is to compare with the real data. Also, it is not fully clear that this is really true even according to their results. For example, the ODE prediction and stochastic results of the number of cells infected with mutants (Figure 1) are not too different; only a couple of cells difference are observed in most of the cases except for small R_0 in advantageous mutation case. So, I am not sure how we can say that they are indeed different. Similarly, the time until mutant generation and number of infected cells (Figure S3) are almost the same in the deterministic and stochastic case (2.6 vs. 3 days, 4.8 days vs 5.6 days, 3.5×10^3 vs 1.4×10^4, and 3.5×10^6 vs 4.3×10^7). It is not convincing to conclude they are different and deterministic results are not correct. The authors may want to just focus on the hybrid method they developed and produce results, without claiming that hybrid is better than deterministic. Basically, they both have different purposes for modeling.

We agree with the referee that both ODE and stochastic modeling have their advantages and disadvantages. The ODE modeling is very intuitive and provides excellent insights into the dynamics. Stochastic models are much harder to implement, slow to run (thus we developed our hybrid method), but they contain more information about evolutionary dynamics. In particular, stochastic modeling allows studies of distributions (such as mutant number distributions and the distribution of generation times). Also, stochastic models can describe the number of mutants at a given population size, or the time of mutant generation, which are not clearly defined in the continuous ODEs.

In the new version of the manuscript we added a discussion of these points. In particular, we avoided projecting qualitative/evaluative statements about the two modeling approaches, which are successfully used by many researchers from different groups. At the same time, we felt that it was important to provide a quantification of the differences in predictions obtained by the two methods. It is true that in some regimes,

the difference is hardly noticeable, while for other parameters the results are significantly different. Giving a range of such results will allow the reader to get a better idea of necessity and applicability of the (significantly more labor-intensive) stochastic techniques.

4. Figure 3 results: the same value is used for β and γ to compare free-virus case ($\beta = 0$ case) and synaptic case ($\gamma = 0$ case). But, a widely different values of N are used ($N = 11$ and $N = 25$). So, the difference obtained could be because of the different value of N, rather than different mechanism. For fare comparison, the both cases should be simulated for the same value of N. Also, what value of S is used?

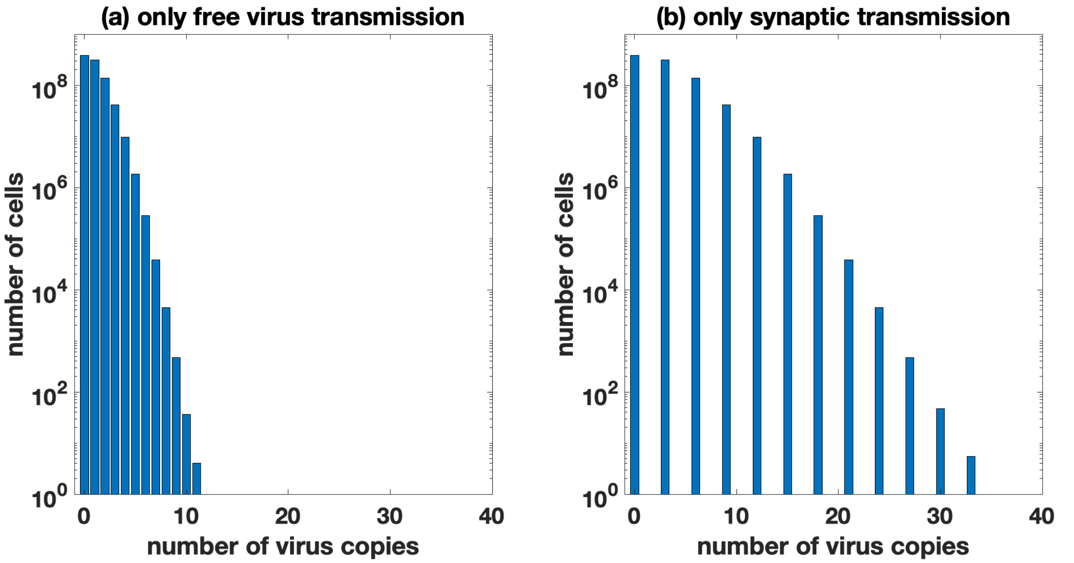

Figure: Multiplicity of infection with different transmission methods.

The parameter N represents the maximum multiplicity of infection, which is the maximum number of viral copies a cell can be infected with. As noted in the Supplementary Information Section 4 for simulations with only free virus transmission, most cells do not become infected with more than 11 copies of virus by the time of peak infection. Similarly, for simulations with only synaptic transmission, most cells do not become infected with more than 25 copies of virus by the time of peak infection. Therefore, for simulations with only free virus transmission, where only one virus copy is transmitted per infection event, we use maximum multiplicity of infection $N = 11$ and for simulations with only synaptic transmission, where S virus copies are transmitted per infection event, we use maximum multiplicity of infection $N = 25$. Increasing N in either case (free virus only versus synaptic only) will have no effect, as almost all cells do not reach this threshold. We use a smaller N for simulations with only free virus transmission for faster computational speed. For all simulations we set $S = 3$ (see Kreger et al. 2021 Virus Evolution PMCID: PMC8117450). These points have been additionally clarified in the caption of Figure 3 and in the new Section 1.3 of the Supplementary Information.

5. How to determine δ or pick reasonable value of δ in Equation (12)?

As described in the main text, δ is the difference threshold between the rate of stochastic extinction of the infection in the stochastic versus hybrid stochastic-deterministic case. Since the rate of stochastic extinction for the completely stochastic case is approximately $\frac{1}{R_0}$, then δ should be small compared to $\frac{1}{R_0}$, i.e. $\delta \ll \frac{1}{R_0}$. The choice of δ then depends on the balance between the fidelity to the fully stochastic simulations (smaller δ) and managing computational expense (larger δ).

It is also important to note that Equation (12) in the main text (which is now Equation (16) in the revised manuscript) only provides a lower bound on the size threshold and that we can confirm that a given population size threshold for the hybrid method is large enough using the ε test described in Section 2.3.1 in the main text and in Rodriguez-Brenes et al. 2019 Journal of Theoretical Biology PMID: 30315815. A remark has been added in Section 2.3.2 to explain the choice of δ.

6. The unit of volume is not given. Is it per milliliter? For HIV, the infected cell population size of 5×10^8 seem too high if it is per milliliter. Total CD4 count is 1000 per microliter, which gives 10^6 per milliliter. Moreover, some study (Stafford, JTB, 2000) considers only 1% of this is target of HIV, which makes only 10^4 cells per millimeter are target cells.

Variables describing cell populations are measured in terms of cell numbers (rather than concentrations). This choice was made because it allows for easy interpretation of stochastic data (which track every cell in small populations). A note has been inserted in Table 1 of the main text to clarify the units.

7. How is basic reproduction number formula in caption of Figure 1 derived? In some studies, the basic reproduction number with wild-type and mutant case appears to be $R_0 = \max\{R_0^w, R_0^m\}$, where R_0^w, R_0^m are the basic reproduction numbers corresponding to wild-type and mutants. How is the expression used here compare with previous studies?

The derivation of the basic reproductive ratio R_0 is now provided in Section 1.4 of the Supplement. As noted by the reviewer, if different strains have different fitness F, then they can have different reproductive numbers, R_0. As we initiate infection with a single cell infected with only the wild-type strain, we set the basic reproductive number $R_0 = R_0^w$, as the basic reproductive number represents the average number of newly infected cells per infected cell at the beginning of infection. A note about this is now also included in Section 1.4 of the SI.

Reviewer: 3

While I understand the desire to present the simplest possible version of the model in the main text, much of the subsequent discussion centres on mutant strains, their fitnesses *etc.* which remain undefined except in the SI, so the net result is less, not more, clear (*e.g.* the caption to Figure 1 only makes sense if one has read the SI).

There should be some reasonable compromise between the level of detail given in the SI and the almost none given in the main text. I also found that the exposition could be improved in places (*e.g.* “a disadvantageous mutant is assumed to have an increased probability that successful infection fails” is a tortuous way of saying “a disadvantageous mutant has a decreased probability of infection”).

We thank the referee for this suggestion. Also following similar advice from Referee 1, in the new version of the manuscript, we present the details of the mathematical modeling in the main text, thus making the mathematical part of the manuscript more coherent.

With the emphasis put on the algorithm (*e.g.* the title) rather than the biological results, it seems to me that the authors should explain/emphasize better how it is novel as compared to the authors’ previous work (cited here as [34]) and other multi-scale approaches that have been used in systems biology. Is it the application to an epidemiological model rather than a cancer model? Further, since the virtue of using thresholds and ODEs is speed (at the price of approximation), it would seem more fair to compare performance to widely-used refinements such as *e.g.* adaptive tau-leaping ([3] is a good summary), rather than Gillespie’s original algorithm from over forty years ago.

We thank the referee for this comment. In what follows we 1) provide a detailed response of why we chose the specific hybrid algorithm and how our current work is novel compared to our previous work, 2) demonstrate that the adaptive tau-leaping method will be much slower than our hybrid algorithm because it requires smaller steps in time (often by several orders of magnitude) and that each step is significantly more computationally expensive, and 3) describe why the tau-leaping method does not make sense for systems with interactions between very small (order of 10^0) and very large (order of 10^9) populations; instead a hybrid stochastic-deterministic setup (which also is more natural to researchers in the field of population dynamics and evolutionary biology) is preferred.

Novelty. Our algorithm is based on our previous work in which the hybrid algorithm was introduced (Rodriguez-Brenes et al. 2019 Journal of Theoretical Biology, cited previously as [34]), but the application to our epidemiological model is novel and presents with many challenges. In our virus dynamics model, we have a system of $\binom{N+2^k}{2^k}$ subpopulations, where k represents the number of point mutations that can occur and N is the maximum multiplicity of infection. When k and/or N is large, this results in a very large system, with a huge number of reactions (i.e. if $N = 25$ and $k = 2$ we have 23,751 subpopulations/differential equations, each including many reactions). The reactions in this model are uninfected cell generation, cell death, and infection of target cells. If we include only free virus transmission, the number of infection events is the number of strains multiplied by the number of subpopulations eligible to be infected, but when synaptic transmission is included, there are many more infection events,

which is correlated with the number of ways to partition S into 2^k non-negative integers that sum to 1,2,…,S. Furthermore, we also have continued persistence of both small (order of 10^0) and large (order of 10^9) populations that interact with each other, which generally implies that tau-leaping will not be efficient compared to the basic Gillespie algorithm (Gillespie 2007 Ann. Rev. Phys. Chem, Cao and Petzold 2008 Comput. Methods. Appl. Mech. Eng). This coexistence of both small and large interacting populations is a strong motivating factor to choose our hybrid algorithm, which explicitly treats large populations deterministically and small populations stochastically.

Furthermore, we also introduce a novel analytic lower bound on the size threshold in the case of virus dynamics models (described in Section 2 of the main text). The size threshold is an extremely important parameter in the algorithm, and our calculation gives a reasonable estimation that does not require extensive and potentially expensive testing of different size thresholds beforehand. The *a priori* determination of the size threshold for the algorithm is subject to ongoing work.

In the new version of the manuscript, we have included a more in-depth discussion on other hybrid approaches including tau-leaping and multi-scale methods in the main text (see Section 2.3). We also included a more detailed description of our work compared to our previous paper (Rodriguez-Brenes et al. 2019 Journal of Theoretical Biology, cited previously as [34]). We have also provided more detail on the methodology and selection of our hybrid algorithm, and how it differs compared to approaches used in physics and chemical kinetics.

Comparison with tau-leaping method. The tau-leaping method/adaptive tau-leaping method measures how many times a given reaction “fires” in a given time interval $[t, t + \tau)$. τ is the step size (either constant or variable in the adaptive framework) which is usually determined as the largest value such that the *leap condition* is satisfied, which requires that no reaction propensity function changes by a “significant” amount in the interval $[t, t + \tau)$. If the number of “firings” of each reaction in the interval $[t, t + \tau)$ is much greater than 1, then the adaptive tau-leaping method will result in a significantly faster stochastic simulation (compared to the basic Gillespie algorithm, which performs each reaction sequentially). Without fully implementing the tau-leaping method, it is possible to show that it is significantly slower than the hybrid method used in our paper. The argument consists of two parts.

(A) We first show that updating the system at each step is less computationally expensive for the hybrid method than for the tau-leaping method. Comparing a stochastic update to a deterministic step using the differential equations for our model, it is straightforward to see from the program code that the deterministic step involves fewer floating point operations compared to a stochastic update. One contributing factor for this is the number of reactions (which is quite large as described above), and that the stochastic updates treat all of these reactions individually. In addition to fewer operations, the deterministic step does not require the generation of any random numbers (which is computationally expensive), whereas the stochastic update step

(either basic Gillespie or tau-leaping method) requires a random number to be generated for *each* reaction channel for each time interval. Therefore, if for instance the hybrid method and tau-leaping method used the same step sizes (i.e. the system gets updated at exactly the same time points $t = 0, t_1, t_2, t_3, \ldots$) for both methods, then the hybrid method will run faster because it involves less operations (and less expensive operations such as pseudo-random number generation) at each step.

The tau-leaping method is also much more operationally expensive (again if updated at the same time points) than just the basic Gillespie method, because of the additional operations needed to calculate τ and confirm it satisfies the leap condition.

Next, we show that the step sizes for tau-leaping will not be larger than the steps for hybrid:

(B) Without fully coding the tau-leaping method, we can calculate if different values of τ satisfy the *leap condition*. One such method is Equation (3) from (Gillespie and Petzold 2003 Journal of Chemical Physics), which is given by

$$\left|a_j(\mathbf{x} + \boldsymbol{\Lambda}(\tau; \mathbf{x})) - a_j(\mathbf{x})\right| \leq \varepsilon a_0(\mathbf{x}) \quad \forall j = 1,2, \ldots, M \qquad (1)$$

where $0 < \varepsilon \ll 1$ is a prespecified error control parameter and a_j is the propensity of the jth reaction. Another method is Equation (17) from (Cao et al. 2006 Journal of Chemical Physics), which is given by

$$\Delta_\tau a_j(\mathbf{x}) \leq max\{\varepsilon a_j(\mathbf{x}), c_j\} \quad j = 1,2, \ldots, M \qquad (2)$$

We set $\varepsilon = 0.1$. In order to demonstrate that the maximum τ allowed by the *leaping condition* is smaller than the deterministic step size (denoted by h), we find that using $\tau = h$ violates both Equations (1) and (2) at all arbitrarily tested points we used $(t = 0.001, 0.01, 1, 2, 5)$. This implies that τ must be smaller than the deterministic step size, that is, hybrid steps are larger (in simulated infection time) than tau-leaping steps.

To summarize: (A) shows that hybrid steps are faster (in computer time) than tau-leaping steps, (B) shows that hybrid steps are larger than tau-leaping steps (in simulated infection time), so (A) and (B) together imply that the hybrid method is faster (less computer time to get to larger simulated infection time) than tau-leaping.

The tau-leaping method is not a good choice for our system. The *leap condition* is best satisfied if all of the reaction propensity functions are large (Gillespie 2007 Ann. Rev. Phys. Chem). However, in our system, as described above, we have the continued persistence of both small and large populations that interact with each other. We begin with 10^9 uninfected cells and a single infected cell. In the timespan under consideration, the number of infected cells with grow to the order of 10^8 cells, however, the total number of infected cells will be subdivided into many subpopulations of infected cells, and we will always have many small populations (< 10) if k and N are sufficiently large. Therefore, measuring how many times a given reaction "fires" in a specified time interval when it affects populations that are of the order of 10^0 cells and other populations of 10^9 cells does not make much sense. This is because small fluctuations in small populations can have a large effect on the evolutionary dynamics of the

infection, but small fluctuations in sufficiently large populations (such as uninfected or highly prevalent cells that are singly infected with the wild type virus) often have very little effect on the dynamics.

Instead, multi-scale methods make more sense for systems with populations of vastly different scales, and so we implement our hybrid method where large populations are treated deterministically (which is both more computationally efficient and makes sense because small fluctuations in large populations have little effect) and small populations are treated stochastically (because small fluctuations in small populations can have large effects which are not captured by deterministic models).

In conclusion, the tau-leaping method is not an optimal choice for our system, and it is also clearly slower than our hybrid method. For these reasons, we did not implement it fully in this paper, but instead, provided a comparison with the conventional Gillespie method. These points have now been included in the Supplementary Information, see new Section 2.4.

The importance of simultaneous synaptic transmission for most evolutionary effects observed in the modelling call into question the well-mixed assumptions made here; it seems like more spatially explicit model (which in turn may not be amenable to ODE approximation due to small neighbourhood sizes) with synaptic transmission to nearby cells might not arrive at similar conclusions. A spatial model is mentioned in the discussion, but it would be useful to really understand how robust the conclusions are to mass-action assumptions.

We agree with the referee that this is an interesting and important question. Further work (described in the conclusion) is in progress and is important, as transmission might have spatial effects in different tissues. Whether spatial effects are important drivers of in vivo HIV dynamics, however, remains an open question. While the formation of virological synapses likely occurs with neighboring cells, imaging studies have pointed out that CD4 T cells move around in the lymphoid tissue with fast rates, which could reduce the importance of spatial effects. Nevertheless, we have investigated spatial aspects of viral transmission in our recent work [Kreger, Komarova, Wodarz (2020) Journal of the Royal Society Interface], where a spatial agent-based model of viral transmission (both free-virus and synaptic) was implemented. Creating a hybrid model of the kind described here, but in a spatial setting, is a subject of future research. We have now briefly discussed this issue in the revised manuscript.

Finally, I would also like to bring to the authors' attention some previous (theoretical) work on multi-scale analysis of stochastic models, in particular [2], which illustrates how one can identify scales and approximations for a viral kinetics model. One of the authors (Tom Kurtz) has decades of work on the subject of stochastic chemical kinetics and approximations, including a recent book [1] which gives a good overview of the subject and an ample bibliography.

We thank the reviewer for bringing our attention to these important references and have included a more detailed discussion on other hybrid approaches including tau-leaping and multi-scale methods, see Section 2.3 in the revised main text.

We first describe why we turn to a hybrid algorithm, which is because as the population size increases the average time step in the Gillespie method decreases, which results in a very computationally inefficient method. In particular, we turn to the method developed in our previous work (Rodriguez-Brenes et al. 2019 Journal of Theoretical Biology, cited previously as [34]), which was specifically developed to handle large population dynamic models where stochastic interactions that effect both very small and very large populations exist (such as random and rare mutation) in an intuitive and computationally efficient way.

We then describe how our hybrid method is based on work in the field of chemical kinetics, which arose from the necessity to simulate systems with a large number of reactants (and so Gillespie is computationally inefficient). Especially in the field of physical chemistry, many innovative computational algorithms have been developed to simulate stochastic systems, including the next reaction method and tau-leaping/adaptive tau-leaping methods. Here, large computational advantages over the Gillespie method are feasible because of the ability to make large time steps through measuring how many times each stochastic reaction “fires” in the given time interval. However, the existence of both small and large populations of importance generally implies that methods such as tau-leaping will be inefficient and furthermore, measuring how many times a reaction “fires” in a given time interval is somewhat counterintuitive when subpopulations vary over several orders of magnitude (Gillespie 2007 Ann. Rev. Phys. Chem, Cao and Petzold 2008 Comput. Methods. Appl. Mech. Eng). To this end, there has also been a focus on the development (as well as mathematical analysis) of novel hybrid stochastic-deterministic approaches, including many different mutli-scale methods (such as fast-slow methods) that are designed to simulate systems that contain different time, size, and spatial scales.

Much important work has also been done on the mathematical properties and analysis of stochastic multi-scale models, including in [Ball et al. (2006); Anderson & Kurtz (2015); Bertram & Rubin (2017); Kelly & Vanden-Eijnden (2017)]. In particular, Ref. [Anderson & Kurtz (2015)] provides detailed mathematical justification and motivation for the use of stochastic continuous-time Markov chains in simulating chemical networks, as well as analytic approaches for model approximation/reduction techniques for complex systems. Ref. [Ball et al. (2006)] provides a theoretical foundation for a class of stochastic models, specifically where some populations and/or reaction propensities vary in size over several orders of magnitude. One such example is the use of a pre-existing model of a cell’s viral infection to demonstrate the power of model dimensionality reduction techniques for mathematically analyzing complex networks. Specifically, a combination of averaging and law of large number arguments is used on the viral dynamics model (which overall has three populations and six stochastic reactions) to 1) show that the ``slow” component of the model can be approximated by a

deterministic equation, 2) characterize the asymptotic distribution of the ``fast" components, and 3) derive important quantities such as the probability a single virus successfully infects a cell and the expected time until the establishment of an infection. These such arguments explicitly provide theoretical justification for the identification of different scales in multi-scale analysis of stochastic models.

While similar model reduction approaches and algorithms are often used in the field of physical chemistry, they are less common in the fields of population dynamics and evolution, as they can rely on theoretical physical concepts such as Langevin's equation. In this paper, we choose to implement the hybrid methodology described in [Rodriguez-Brenes (2019)], which partitions the system into a large and small component through the use of an explicit size threshold. This is because our evolutionary system under consideration contains a large overall population size and number of reactions, random and rare mutation events, and the simultaneous existence of both large and small populations of importance. The use of the hybrid method allows us to run computer simulations of the system at a reasonable computational expense.

Other comments & questions:
It would benefit potential readers not familiar with HIV dynamics to briefly define/distinguish free *vs.* synaptic transmission when they appear at the top of page 2 (incidentally, adding line numbers via the package *lineno* would simplify life for your reviewers).

The following sentence in the introduction introduces synaptic transmission: "*In this process multiple viruses are likely transferred from the source cell to the target cell (this is in contrast to free virus transmission, where offspring virus is released into the extracellular environment, typically leading to the infection of the target cell with a single virus). The process of synaptic transmission has been well documented experimentally [23, 8], and experiments with humanized mice indicate the importance of this process in vivo [34].*

Also, line numbers have been added in the new version of the file.

Please define S in the paragraph preceding equations (1) – (5). Any reader familiar with epidemiological models recognizes λ, β and γ, and can thus understand these without need to refer to the table. That is not true of S, which is an important component of the model structure.

Parameter S is now defined right after ODEs (1-5), as well as in Table 1.

In equations (2) and (4), writing $Z(-x_i)$ and $Z(x_{i-S})$ give the reader that the function Z is being evaluated at $x_i$and x_{i-S} respectively (and by extension, Z is being evaluated at the quantities in brackets elsewhere). It would be clearer to write $-\gamma Z x_i$ and $+\gamma Z x_{i-S}$.

We thank the reviewer for pointing this out, this has been changed.

The extinction probability $\frac{1}{R_0}$ for a novel strain (used here to determine the threshold *M*) is only valid at the disease-free equilibrium (all cells susceptible); how does this affect the choice of thresholds for mutant strains?

In the revised version of the manuscript, we have added the following text: Note that M is a lower bound, and the calculation above is based only on the dynamics of the wild type strain, without taking into account any information on the mutant parameters. Therefore, depending on the details of the model (such as the number and type of mutant strains), it is possible that a larger M is needed to get accurate descriptions of mutant dynamics.

“This is because at time t_N, while the average trajectory reaches size N, some stochastic trajectories at that time will be lower and some higher than N.” The trajectories are multi-dimensional; it would be better here to speak of the number infected.

We thank the reviewer for pointing this out, this has been fixed.

Results are presented for the exponential growth phase. Do the authors account for the possibility that the number infected will fall below the threshold as a part of the boom-bust dynamics inherent in the model?

Yes, we do account for the possibility that the number of infected cells (as well as the number of each subpopulation of cells) can fall below the threshold at any point in the model. Individual subpopulations of cells can fluctuate (sometimes above the size threshold and sometimes below) even in the context of exponential growth for the total number of infected cells, so this is especially important. When a cell subpopulation that was previously large falls below the threshold M, it is again treated stochastically by the hybrid stochastic-deterministic algorithm. The classification of small versus large populations is re-checked at every step in the algorithm. (We have clarified this in the text).

Figure 2 displays histograms, not distributions (the latter are normalized).

We thank the reviewer for pointing this out and have changed references in Figure 2 in the main text and similar figures in the Supplementary Information to denote that they display histograms and not distributions.

Supplementary Information

What mutation is assumed? I wonder how likely it is that multiple sites segregate simultaneously as opposed to sequentially?

The mutation rate of 3×10^{-5} (typical for HIV) is used. The model is general enough to account for simultaneous as well as sequential mutational events.

For the event notation, please indicate that the triples indicate outcomes for the three viruses. Writing the sets of events in brackets gives the impression of quadruples; ($* * *$, $* * X$, $*XX$, XXX), ($* * 0$, $*X0$, $XX0$, $*00$, $X00$, 000) would be perhaps more clearly indicated by $\{* * *, * * X, *XX, XXX\}$ and $\{* * 0, *X0, XX0, *00, X00, 000\}$.

In the new version of the manuscript we used the curly brackets, as suggested by the referee.

Equation (2): I fail to see what is gained by representing a polynomial as a hypergeometric function. Is this done for the purposes of coding?

Yes, this could be beneficial for coding e.g. using *Mathematica.* We have removed this representation from the new version.

I don't know what the phrase "Everything in Equations (14)-(16) is stochastic." is intended to say when applied to an ordinary differential equation. I'm assuming the ODE is meant as a shorthand for the jump rates of a continuous-time Markov chain, but that seems to more confuse than clarify.

This is correct, we were intending to convey that because the cellular subpopulations $x_2(t^*), x_3(t^*), x_4(t^*)$ are all classified as small at time t^*, that their respective ODEs define the jump rates (also referred to as reaction terms or reaction propensities) of the Markov chain, i.e. that these reactions described in the ODEs are included in the stochastic Gillespie step of the hybrid algorithm because $x_2(t^*), x_3(t^*), x_4(t^*)$ are not large. We thank the reviewer for bringing our attention to this poorly phrased sentence and have revised the corresponding text to clarify that the individual terms in these equations are reactions that are treated stochastically by the algorithm.

Figure S1 (b): can you explain the tail behaviour (what looks like a second peak at around 10) in the hybrid simulations?

We thank the reviewer for pointing this out. The tail behavior (what looked like a second peak at around 10 in Figure S1(b) which is now Figure S3(b) in the revised SI) was due to random effects because of a relatively small number of simulations to create the histogram plots (3×10^3 simulations for each plot). We have increased the number of simulations for Figure S1 in the Supplementary Information to 1.5×10^5 simulations and have updated the figure in the Supplementary Information (please see the new Figure S3) as well as included it below. Here, with a larger number of simulations, we see a smoother plot that clearly shows that the number of cells infected with the double mutant is generally decreasing and that there is no second peak. We have also re-verified that for double mutants, the histogram distributions of the cell numbers infected with mutant viruses are not statistically different for a size threshold of $M = 50$ versus $M = 500$ ($p > 0.1$ by the Kolmogorov-Smirnov test). This demonstrates that for $R_0 = 8$, a size threshold of $M = 50$ is sufficiently large enough to provide of good approximation of the fully stochastic dynamics for double mutants.

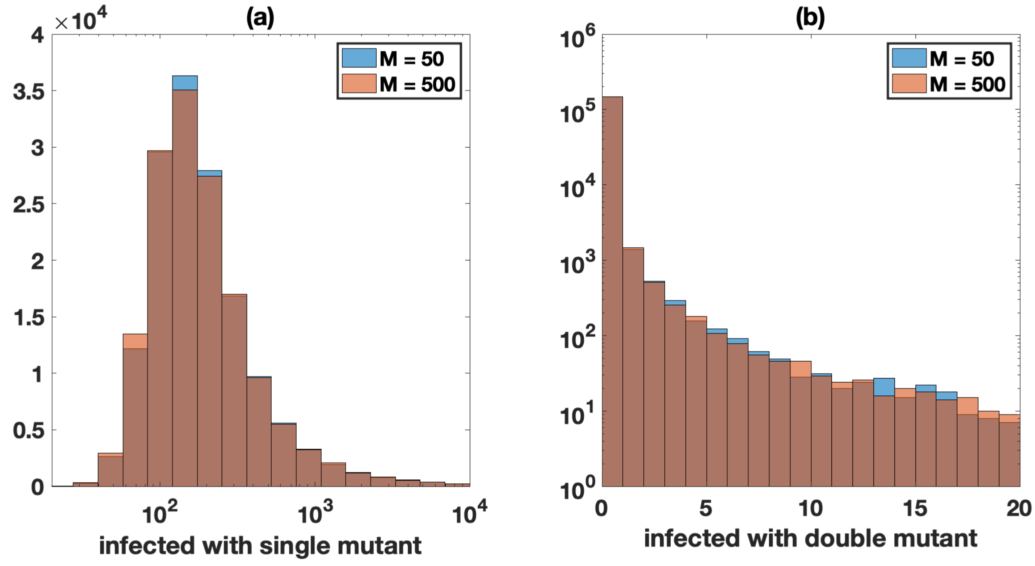

Fig: Updated Figure S1 (which is now Figure S3) with more simulations.
