# Supplementary material for: A hybrid stochastic-deterministic approach to explore multiple infection and evolution in HIV
Source: PLoS Comput Biol. 2021 Dec 22;17(12):e1009713. doi: 10.1371/journal.pcbi.1009713 (PMC8730440; doi:10.1371/journal.pcbi.1009713)
Supplement: Attachment — Submitted filename: Kreger_et_al_v2.pdf [file pcbi.1009713.s019.pdf]

Review of Kreger *et. al.*, *A hybrid stochastic-deterministic approach to explore multiple infection and evolution in HIV*, revision

By and large, the reviewers have addressed my comments and concerns regarding the first version of the paper. The description of the algorithm in SI §2.2, however, continues to obscure rather than illuminates what is essentially a simple algorithm: namely that the number of cells that are uninfected or infected with a single copy of the virus are modelled deterministically via ODEs

$$\begin{aligned} \dot{x}_0 &= \lambda - \beta Z x_0 - d x_0 \\ \dot{x}_1 &= \beta Z x_0 - \beta Z x_1 - a x_1, \end{aligned} \tag{1}$$

whereas the number of cells infected with 2 to 4 virus copies are modelled as continuous time Markov chains (CTMC) with time-dependent jump rates

**Jump**	**Rate**
$x_2 \to x_2 + 1$	$\beta Z x_1$
$x_2 \to x_2 - 1$	$a x_2$
$x_2 \to x_2 - 1$, $x_3 \to x_3 + 1$	$\beta Z x_2$
$x_3 \to x_3 - 1$	$a x_3$
$x_3 \to x_3 - 1$, $x_4 \to x_4 + 1$	$\beta Z x_3$
$x_4 \to x_4 - 1$	$a x_4$

with x_0 and x_1 determined by (1), until the first time that $x_2(t) > \mathcal{M}^*$, after which x_2 is given by

$$\dot{x}_2 = \beta Z x_1 - \beta Z x_2 - a x_2,$$

etc.. Incidentally, such processes (without the changeover between deterministic and stochastic approaches at a threshold) are called piecewise deterministic Markov processes (PDMP), and there is considerable literature on their efficient implementation numerically.
