# Supplementary material for: A hybrid stochastic-deterministic approach to explore multiple infection and evolution in HIV
Source: PLoS Comput Biol. 2021 Dec 22;17(12):e1009713. doi: 10.1371/journal.pcbi.1009713 (PMC8730440; doi:10.1371/journal.pcbi.1009713)
Supplement: Attachment — Submitted filename: Review response.pdf [file pcbi.1009713.s020.pdf]

A hybrid stochastic-deterministic approach to explore multiple infection and evolution in HIV

Author responses and changes

We would like to thank the editors and the reviewers for carefully reading both our original and revised manuscript and providing valuable suggestions and comments to improve the paper. We are glad that the reviewers agree that their comments have been addressed in the revised manuscript.

Below are our detailed responses and changes to the two comments made by the reviewers following their review of the revised manuscript. The reviewers' comments are in blue and our responses are in black. Changes in the main text and supplement are shown in blue font.

Reviewer: 1

The authors have made great improvements to the flow in their revisions, and have responded to my previous questions. I am not sure why the variables with * in Table 1 do not simply have units in the units column instead of the note in the caption, but I assume the authors have a specific reason for this choice.

We would first like to thank the reviewer for critically reviewing our manuscript and for their many helpful suggestions.

The variables/parameters from x_0 down in Table 1 in the main text are unitless. We have updated the format of Table 1 in the main text to include the types of quantitates these variables/parameters are and have removed the * notation so that Table 1 is presented in a clearer way.

Reviewer: 2

Authors have addressed all my comments. I thank them for the updated work. I do not have any further comments and recommend for publication in PLOS Computational Biology.

We thank the reviewer for their comment above and for critically reviewing our manuscript. Their many insightful suggestions during the initial review led to many improvements in the revised manuscript.

Reviewer: 3

By and large, the reviewers have addressed my comments and concerns regarding the first version of the paper. The description of the algorithm in SI 2.2, however, continues

to obscure rather than illuminates what is essentially a simple algorithm: namely that the number of cells that are uninfected or infected with a single copy of the virus are modelled deterministically via ODEs

$$\begin{aligned} \dot{x}_0 &= \lambda - \beta Z x_0 - d x_0 \\ \dot{x}_1 &= \beta Z x_0 - \beta Z x_1 - a x_1 \end{aligned} \quad (1)$$

whereas the number of cells infected with 2 to 4 virus copies are modelled as continuous time Markov chains (CTMC) with time-dependent jump rates

Jump	Rate
$x_2 \to x_2 + 1$	$\beta Z x_1$
$x_2 \to x_2 - 1$	$a x_2$
$x_2 \to x_2 - 1,\ x_3 \to x_3 + 1$	$\beta Z x_2$
$x_3 \to x_3 - 1$	$a x_3$
$x_3 \to x_3 - 1,\ x_4 \to x_4 + 1$	$\beta Z x_3$
$x_4 \to x_4 - 1$	$a x_4$

with x_0 and x_1 determined by (1), until the first time that $x_2(t) > M^*$, after which x_2 is given by

$$\dot{x}_2 = \beta Z x_1 - \beta Z x_2 - a x_2$$

etc.. Incidentally, such processes (without the changeover between deterministic and stochastic approaches at a threshold) are called piecewise deterministic Markov processes (PDMP), and there is considerable literature on their efficient implementation numerically.

We would first like to thank the reviewer for carefully reviewing our manuscript and for their helpful suggestions, both in the initial round of review and for the revised manuscript.

We have revised the description of the algorithm in Section 2.2 of the Supplementary Information. We now include the jump/rate table provided by the reviewer in order to explicitly show the stochastic reactions and their rates, and we have also additionally clarified the description of the algorithm in this section.

As the reviewer notes, our stochastic-deterministic algorithm is similar to piecewise deterministic Markov processes (PDMPs), where a deterministic motion/process is modified to include discrete stochastic Markov jumps. We have added a discussion comparing our algorithm with PDMPs in Section 2.5 of the Supplementary Information. PDMPs have been used to model a wide variety of biological systems including processes in population dynamics, genetics, and physical chemistry. There has also been much work on the efficient numerical implementation of PDMPs, as simulations can often be computationally expensive and difficult. In particular, simulations of

Hodgkin–Huxley type models have been greatly improved by different PDMP simulation methods.

Our algorithm is different from standard PDMPs because the different reactions in the system bounce back and forth from deterministic to stochastic implementations based on the size of the populations involved in the reactions and the size threshold M. While simulation methods from PDMPs are not directly applicable to our algorithm, both theoretical analysis of PDMPs and methods to efficiently implement them are likely important for future work in virus dynamics and hybrid stochastic-deterministic algorithms.
